# Emerging Biomarkers and Determinants of Lipoprotein Profiles to Predict CVD Risk: Implications for Precision Nutrition

**DOI:** 10.3390/nu17010042

**Published:** 2024-12-27

**Authors:** Catherine J. Andersen, Maria Luz Fernandez

**Affiliations:** 1 Department of Nutritional Sciences, University of Connecticut, Storrs, CT 06269, USA; catherine.andersen@uconn.edu; 2 School of Nutrition and Wellness, University of Arizona, Tucson, AZ 85712, USA

**Keywords:** biomarkers, cardiovascular disease, dyslipidemias, genetics, lifestyle, omics, gut microbiota

## Abstract

Biomarkers constitute a valuable tool to diagnose both the incidence and the prevalence of chronic diseases and may help to inform the design and effectiveness of precision nutrition interventions. Cardiovascular disease (CVD) continues to be the foremost cause of death all over the world. While the reasons that lead to increased risk for CVD are multifactorial, dyslipidemias, plasma concentrations of specific lipoproteins, and dynamic measures of lipoprotein function are strong biomarkers to predict and document coronary heart disease incidence. The aim of this review is to provide a comprehensive evaluation of the biomarkers and emerging approaches that can be utilized to characterize lipoprotein profiles as predictive tools for assessing CVD risk, including the assessment of traditional clinical lipid panels, measures of lipoprotein efflux capacity and inflammatory and antioxidant activity, and omics-based characterization of lipoprotein composition and regulators of lipoprotein metabolism. In addition, we discuss demographic, genetic, metagenomic, and lifestyle determinants of lipoprotein profiles—such as age, sex, gene variants and single-nucleotide polymorphisms, gut microbiome profiles, dietary patterns, physical inactivity, obesity status, smoking and alcohol intake, and stress—which are likely to be essential factors to explain interindividual responses to precision nutrition recommendations to mitigate CVD risk.

## 1. Introduction

Biomarkers are indicators of normal and pathological biological processes. The concept of biomarkers is not novel, as ancient civilizations utilized biomarkers to assess disease state. For example, the degree of injury was assessed by monitoring heart pulse [1]. The first determination of blood pressure was recorded as early as 1732 by introduction of a probe into a horse [2]. The utilization of measuring specific components in blood started from the 1800s and evolved to the determination of biomarkers that are currently used [3]. Nowadays, biomarkers are important tools in diagnosing disease or predisposition to disease, and they can be detected in blood, saliva, stool, and urine, as well as in tumors and tissue samples collected from biopsies, pap smears, and more [3]. With the increasing value of utilizing precision nutrition to provide accurate guidance related to diet, and the greater understanding of diet–gene interactions, biomarkers have acquired a more substantial role in predicting chronic disease. Some of these biomarkers are already utilized in clinical practice and some are used as surrogate markers of the effectiveness of drugs [4]. Due to the explosion of “omics”, defined as the collective characterization of the quantification of biological molecules, the discovery of biomarkers that can be associated with chronic disease has dramatically expanded [3]. However, while some biomarkers are well established and used as diagnostic tools for certain chronic conditions, some are still in the process of being validated. To improve the quality of reporting and diagnostic accuracy, the STARD (Standard for Reporting of Diagnostic Accuracy) has been formed [5]. However, further research is continuously needed to evolve and improve both standard and emerging biomarkers, and to lower the costs and barriers in the implementation of measurements of these biomarkers in clinical practice, particularly those requiring more advanced technologies.

Cardiovascular disease (CVD) is the leading cause of death worldwide. In 2022, CVD contributed to 702,880 deaths with a cost of 252.2 billion dollars [6,7]. Accordingly, significant research over the last decade has aimed to identify and improve clinical and translational biomarkers to better predict CVD risk and assess the effectiveness of CVD-related therapeutics. While research has identified various serum proteins, metabolites and clinical cell markers that can contribute to the pathophysiology and risk prediction of CVD risk, plasma lipid and lipoprotein measures are the most established and commonly used indicators of CVD risk and will be the focus of this review [8,9,10,11]. Importantly, plasma lipid and lipoprotein biomarkers of CVD risk not only include standard measures of serum concentrations of plasma lipids and lipoprotein subfractions in clinical settings, but also more recent measures of lipoprotein modification and function including efflux capacity, inflammatory, and antioxidant activity, which have been developed in research settings utilizing ex vivo translational techniques and enhancing predictive power for CVD [12,13,14,15]. In addition, the advancement of omics-based approaches, including plasma and lipoprotein fraction-specific characterization of lipidomic and proteomic composition not only provides greater insight into lipoprotein functionality within the context of CVD pathophysiology, but may help to identify regulatory pathways by which lipoprotein metabolism and function can be targeted to enhance CVD therapeutics [16,17,18,19]. However, it is important to note that the strength of evidence and validation of these emerging approaches is still on-going and may face steep barriers to implementation in clinical settings due to current high costs and a lack of standardized automated testing protocols, although work is ongoing to address feasibility of these methods [19,20].

In order to design effective CVD therapeutics for diverse populations, it is critical to understand the determinants of plasma lipid and lipoprotein profiles. In line with differences in CVD risk regarding age, sex, and race/ethnicity groups, differences in plasma lipid and lipoprotein profiles differ across populations, and are thought, in part, to drive differences in disease risk [21,22,23,24,25]. Further, gene variants, including *apolipoprotein E* (*APOE*) isoforms, and single-nucleotide polymorphisms in genes encoding the LDL-receptor (LDL-R), apolipoprotein B (apoB), and proprotein convertase subtilisin/kexin type 9 (PCSK9) may result in severe hypercholesterolemia and hypertriglyceridemia [26,27]. In addition, adverse plasma lipid and lipoprotein composition and function profiles are observed in populations with specific lifestyle characteristics including unhealthy diets, alcohol consumption, smoking, obesity, physical inactivity, and stress, as well as those with gut microbiome dysbiosis; thus, many pharmaceutical and lifestyle recommendations aimed at reducing CVD risk target these factors [28,29]. However, while various dietary patterns and recommendations have been put forth by agencies aimed at reducing CVD risk and have shown to be generally effective including the Mediterranean Diet, Dietary Approaches to Stop Hypertension (DASH), the American Heart Association diet and the ketogenic diets, these dietary recommendations tend to be universal for all populations, with the problem that effectiveness varies across populations [30,31,32,33]. Thus, the emerging field of precision nutrition aims to individualize recommendations to specific patients or populations, considering many of the determinants described above. It is important to note, however, that research validating precision nutrition approaches is limited, and many researchers are working to develop and standardize how to effectively design and interpret precision nutrition results in a way that is meaningful and can reasonably be implemented in clinical settings [34,35].

**Figure 1:**
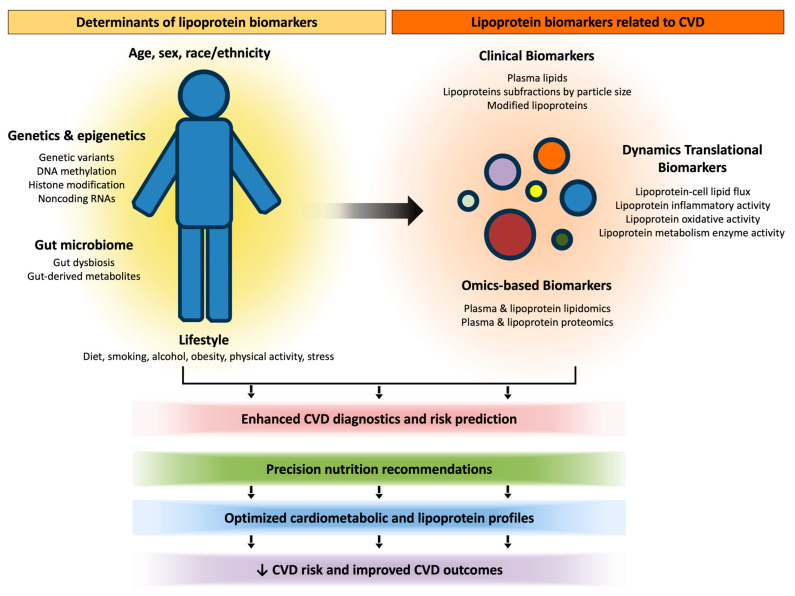
**Determinants of lipoprotein biomarkers for consideration of precision nutrition strategies to reduce CVD.** Lipoprotein biomarkers that are relevant to CVD diagnosis and risk assessment include those that are standard in clinical settings, in addition to dynamic translation measures of lipoprotein function and metabolism, as well as omics-based plasma and lipoprotein-specific measures of lipid and protein profiles. Importantly, demographic, genetic and epigenetic, metagenomic and metabolomic, and lifestyle factors are known to influence lipoprotein biomarkers. Thus, consideration of these factors may be critical in the design of effective precision nutrition strategies aimed at reducing CVD. CVD: cardiovascular disease.

In this review, we outline the diverse methodologies used to identify predictive lipoprotein biomarkers within the context of CVD risk, including lipidomic, proteomic, metagenomic/gut microbiome, genetic, and epigenetic approaches that aim to better characterize how interindividual variability in lipoprotein composition and metabolism contributes to differences in CVD risk. The biomarkers obtained by standardized and emerging techniques will be discussed with an emphasis on their strengths and weakness for diagnostic and predictive CVD risk potential. In addition, since various lifestyle factors play a major role in the development and progression of CVD [28,29,36], their contribution to the regulation of lipoprotein biomarkers and CVD risk will be addressed in this review. Finally, we highlight future recommendations for how lipoprotein profiles, lifestyle and genetics factors, and omics-based approaches can be utilized for precision nutrition interventions. A summary of topics discussed in this review of presented in Figure 1.

## 2. Traditional Clinical Biomarkers Used to Characterize Lipoprotein Profiles and Predict CVD Risk

### 2.1. Plasma Lipid Panels

The measurement of plasma lipids—specifically plasma LDL-cholesterol (LDL-C) concentrations—is the most universal approach to evaluating lipoprotein biomarkers to assess risk for atherosclerosis and CVD. The use of these biomarkers in clinical practice can be attributed to the ability to measure these markers in blood samples collected in routine clinical practice, the availability of rapid, standardized, and relatively low-cost reagents and equipment to perform the measurements across many different clinical laboratories, the coverage of these measures by medical insurance carriers, acceptability and relative ease of interpretation by healthcare professionals, and a large body of research from observational and intervention trials that demonstrate associations between plasma lipids and LDL-C concentrations and CVD risk, progression, and events [37,38,39,40,41,42,43,44,45,46,47]. However, as with many biomarkers, there are limitations and exceptions to plasma lipids-CVD associations, since plasma lipid measures cannot comprehensively depict the highly dynamic and systemic processes involved in lipoprotein metabolism and cellular interactions. Accordingly, it has been known for some time that individuals with normal LDL-C levels have shown occlusion in the coronaries measured by CT scans or have even experienced myocardial infarctions [37], indicating that other factors are involved. The discovery in 1997 that small dense LDL particles are more susceptible to oxidation and can be the beginning of plaque accumulation has now helped acknowledge that the size of the LDL particle is an important factor to consider for diagnosis of CVD risk [38]. This knowledge strongly suggests that measuring plasma LDL-C alone does not provide a complete picture of CVD risk. Similarly, low plasma concentrations of HDL cholesterol (HDL-C) have traditionally been considered problematic while elevated concentrations are protective [39]. However, again this concept has also been challenged due to the recognition that HDL can be pro-atherogenic depending on its functional activity and other proteins that it carries [40]. Accordingly, a recent large-scale study found that both low and elevated HDL increased risk of mortality [41]. HDL has a number of key functions that protect against CVD risk including its antioxidant, anti-inflammatory, and anti-coagulant properties, as well as its function of removing cholesterol from the vessel walls in the process known as reverse cholesterol transport (RCT). However, HDL can become dysfunctional through the action of the pro-inflammatory enzyme myeloperoxidase that causes oxidative modification and nitrosylation of apolipoprotein A-I (apoA-I). This dysfunctionality leads to impaired ATP-binding cassette transporter A 1 (ABCA1)-mediated macrophage transport, as well as the activation of inflammatory pathways, leading to an increased risk of coronary artery disease (CAD) [42]. Thus, understanding the features of dysfunctional HDL in clinical practice might lead to new diagnostic and therapeutic approaches to atherosclerosis [34]. However, despite the limitations and new research findings that challenge use of standard LDL- and HDL-C measures for predicting CVD risk, the ease and low cost of performing these measures, and the many earlier studies demonstrating their utility, continue to support their use in clinical practice.

Aside from assessing plasma concentrations of LDL-C and HDL-C independently, the LDL-C/HDL-C ratio has been recognized as a stronger biomarker with higher predictive potential because it better reflects the two-way traffic of cholesterol entering and leaving the arterial intima [43]. Plasma concentrations of apolipoprotein apoA-I and apoB have also been shown to predict CVD since they provide information related to the number of HDL and apoB-containing lipoprotein particles, including VLDL, IDL, and LDL [44]. However, they are less routinely used for clinical diagnostics, whereas LDL-C/HDL-C ratio measures are standard in annual preventative and diagnostic clinical assessments due to the relatively lower costs and greater ease of calculation from standard measurements [45].

In addition to cholesterol-based measures, elevated plasma triacylglycerols (TAGs) are a well-recognized factor associated with CVD risk since TAG-rich lipoproteins such as VLDL may infiltrate the adipose tissue and cause inflammation, or these same lipoproteins can be taken up by the endothelial wall and, because of their high content of cholesterol, they can also induce atherosclerosis [46]. Accordingly, the TAG/HDL-C ratio has also been identified as an indicator of cardiometabolic risk in young adults [47].

### 2.2. Plasma Lipoprotein Subfractions by Particle Size and Number

In addition to measuring plasma concentrations of lipoprotein-associated lipids, plasma concentrations of specific lipoprotein class subfractions of distinct sizes are available for assessment in clinical settings and are known to predict CVD risk and CAD severity, as seen in a wide range of studies [48]. While lipoprotein subfractions have historically been analyzed in research settings utilizing centrifugation and gel electrophoresis-based approaches, nowadays, there is greater utilization of ion mobility or nuclear magnetic resonance-based methods, where information regarding total VLDL, IDL, LDL, and HDL particle number, average particle diameters, and concentrations of small, medium, and large particles can be quantified [49]. It is important to note that due to cost, these techniques are more commonly used for research purposes or in high-risk clinical populations, although partial or full coverage of costs is often available from Medicare or private insurance providers in the U.S. [50].

As noted above, plasma concentrations of small LDL are more susceptible to oxidation and arterial uptake and deposition, leading to atherosclerotic foam cell formation, and are known to be independent and stronger predictors of CVD than serum lipids in individual who have both low and very high risk of CVD [51]. Similarly, a higher number of total LDL particles in plasma is associated with increased CVD risk. Conversely, higher average LDL particle size and increased concentrations of large LDL particles are inversely associated with CVD risk, which is thought to be due to greater resistance of the particle to oxidation and modification, although this association varies across studies [48].

In addition to LDL, VLDL particle size and diameter provide predictive power to CVD risk scores. Larger VLDL particles are reflective of more TAG-rich particles that are more cytotoxic to endothelial cells, promote adverse arterial remodeling, and are prone to atherogenic changes in the apolipoproteins carried by the particle [52]. Accordingly, plasma concentrations of large VLDL are positively associated with coronary artery calcification [53,54], in addition to increased overall risk of CVD and type 2 diabetes [55,56].

Finally, plasma concentrations of total HDL particles, HDL of distinct size categories, and average HDL particle size provide further insight into CVD risk prediction. The most consistent associations to CVD risk are found in the large HDL and the average HDL particle size. Large HDL is reflective of more cholesterol-rich particles, which positively correlate with plasma HDL-C concentrations. Accordingly, higher plasma concentrations of large HDL particles and greater average HDL particle size are often found to be negatively associated with CVD risk [57]. However, it is important to note that the HDL-CVD risk associations are less consistent than the associations between CVD risk and other lipoprotein subfractions, in line with more recent U-shaped relationship between HDL-C and CVD risk [41]. This is likely due to the relatively greater complexity of HDL functions and composition. HDL particles of different sizes are also known to have different functional properties, including varied specificity for mediating cellular lipid as well as exhibiting varied antioxidant and anti-inflammatory activities, which is often attributed to the biophysical and structural properties of different HDL particle sizes and the remodeling of HDL-associated proteins, which are being increasingly better characterized in research settings [58,59,60]. Further investigation is warranted to determine whether standard clinical measures of HDL can serve as surrogate and more readily translatable, validated indicators of more complex HDL functions to better leverage clinical HDL measures to predict CVD risk.

### 2.3. Modified Lipoprotein Biomarkers Measured in Clinical Practice

Beyond measures of plasma lipid and particle subfractions by size, clinical laboratories additionally offer services to quantify concentrations of modified lipoproteins in plasma that are known predictors of CVD risk. One of the most established modified lipoproteins that serves as a positive indicator of CVD is lipoprotein(a), or Lp(a), which is an apoB-containing LDL-like particle bound to apolipoprotein(a) [61]. Lp(a) increases the risk of CVD via multiple mechanisms. It is pro-atherogenic by exhibiting LDL-like biological activity, with levels higher than 30 mg/dL considered to be problematic. Lp(a) is additionally pro-thrombotic due to apo(a) structural similarities to plasminogen, leading to competition with plasmin to bind to fibrin and potentially to stroke by impeding the dissolution of blood clots [62]. Lp(a) further exhibits pro-inflammatory activity by servings as the primary carrier of proatherogenic oxidized phospholipids [63]. Concentrations of Lp(a) appear to have a highly genetic component as plasma concentrations are largely driven by hepatic production rates [61,64]; however, some studies in the Netherlands have demonstrated that intake of trans fatty acids increases the concentrations of this lipoprotein [65]. Similarly, clinical laboratories now perform plasma measurements of oxidized LDL—LDL particles with free radical-induced oxidatively damaged lipid and protein components—which has been positively associated with increased risk of CVD events and premature atherosclerosis due to an increased propensity to be scavenged by arterial wall macrophages and promote foam cell formation. Accordingly, oxidized LDL has been shown to be an independent predictor of CVD [15,66]. Together, the inclusion of modified lipoprotein measures may offer better insight into CVD risk profiles and targeted therapeutic approaches.

### 2.4. Summary of Traditional Clinical Biomarkers Used to Characterize Lipoprotein Profiles and Predict CVD Risk

In summary, standard clinical biomarkers of lipoprotein profiles are routinely utilized to predict CVD risk in the general population, in addition to those at higher risk for CVD. A summary of clinical biomarkers, including the methods by which they are commonly measured in clinical and research laboratories, as well as their clinical applicability and predictive strengths and limitations for CVD risk, are highlighted in Table 1. As with all biomarkers, it is important to note that the strength of predictive value is not always equally observed across studies, which may be due to differences in populations, study design, or statistical approaches [67]. Continued research is warranted to further optimize and expand upon the biomarkers available for assessment in clinical settings to better mitigate CVD burden.

## 3. Dynamic Translational Biomarkers of Lipoprotein Metabolism and Function to Predict CVD Risk

Clinical measures of lipoprotein profiles are used routinely to assess risk for CVD; however, translational dynamic measures of lipoprotein functionality and metabolism are becoming more common in research settings. While these approaches are less well established, some studies have found that these mechanistic biomarkers may be more accurate in assessing the risk, diagnosis, and recurrence of CVD-related events [72,93]. In the next paragraphs, we will discuss different methodologies that have been utilized to identify the emerging biomarkers used to assess lipoprotein function and metabolism in relation to CVD.

### 3.1. Measurements of Lipoprotein–Cell Cholesterol Flux

Essential lipoprotein-mediated pathways in the progression or regression of CVD involve lipid exchange between lipoproteins and cells. These pathways include peripheral cell uptake of cholesterol derived from LDL particles and efflux mediated by apoA-I/HDL, in addition to mechanisms of lipoprotein particle clearance as part of RCT [94]. Research-based methodologies and commercial kits have been developed to assess research participant- or patient-derived lipoproteins in their capacity to engage in cellular cholesterol flux ex vivo, utilizing flow cytometry, microscopy, fluorescence, and radiolabeled-based cell assays [95,96,97]. To date, LDL and other apoB-containing lipoprotein uptake assays such as Lp(a) are predominantly used for mechanistic, basic science studies rather than the assessment of an individual’s CVD risk [98,99]; thus, further research is warranted to evaluate whether these measures could offer clinical insight. Alternatively, larger-scale studies have demonstrated that HDL-related efflux measures, including the total cholesterol-accepting capacity of serum, are significant predictors CAD, with greater efflux/cholesterol-accepting capacities being inversely associated with CAD severity and CVD risk. Further, cholesterol efflux capacity shows greater diagnostic potential than HDL-C, since it takes into account one of the most atheroprotective properties of HDL: its capacity to facilitate cholesterol efflux [72,73]. The cholesterol efflux capacity of HDL can be impacted by many HDL characteristics, including the relative distribution of HDL particle size, the presence of HDL-associated proteins, the lipid composition of HDL, and whether HDL has been modified by oxidation. For example, populations with CVD or those individuals who have increased risk of CVD often have HDL with relatively higher TAG content, increased concentrations of pro-inflammatory proteins such as serum amyloid A (SAA) that can displace apoA-I, lower concentrations of the antioxidant enzymes paraoxonase 1/3 (PON1/3) that helps to facilitate stable docking of HDL particle to cells, and greater concentrations of small vs. large HDL particles which may be more prone to oxidation—all factors known to negatively impact total cholesterol efflux from cells [100,101,102]. Despite strong evidence for the use of HDL or serum efflux capacity in predicting and diagnosing CVD, current methods utilizing mammalian cell culture models—which are standard in research settings—may be less feasible in clinical laboratories.

### 3.2. Inflammatory Activity

In addition to mediating cellular lipid exchange, lipoproteins possess pro- and anti-inflammatory activity [93,103]. Similarly to measures of cholesterol flux, the inflammatory activity of lipoprotein can be assessed in research settings by utilizing ex vivo cell culture-based methods and molecular techniques. For example, lipoproteins isolated from plasma or serum can be cultured with mammalian cell lines relevant to CVD including macrophages or vascular endothelial cells in the presence or absence of a pro-inflammatory stimuli, and then the cells and/or culture media can be evaluated for indicators of inflammation, such as the upregulation of pro-inflammatory genes or the secretion of pro-inflammatory cytokines, or by utilizing cocultures with monocytes to determine chemoattractant activity [93,103,104,105,106]. Alternatively, the use of cell-free assays has additionally been reported [107,108]. Using these approaches, researchers have demonstrated that oxidized LDL promotes anti-inflammatory M2 macrophages to shift toward a pro-inflammatory M1 phenotype and increased foam cell formation [109]. In lipoproteins isolated from the plasma of healthy vs. hemodialysis patients—who exhibit an increased incidence of dyslipidemia, accelerated atherosclerosis, and greater risk of CVD-related death—it was observed that LDL from hemodialysis patients had greater pro-inflammatory effects on cultured human aortic endothelial cells, as measured by monocyte chemoattractant activity, whereas HDL from hemodialysis patients was less anti-inflammatory. Interestingly, the addition of the apoA-I mimetic peptide 4F suppressed the pro-inflammatory activity of LDL while enhancing the anti-inflammatory of HDL [110]. Further, improvements in cardiometabolic profiles corresponded to improved anti-inflammatory activity of HDL in CHD patients, with HDL inflammatory activity serving as a more significant differentiator of treatment vs. control groups than HDL-C [93]. Importantly, in a case–control Prevention of Renal and Vascular End Stage Disease (PREVEND) study of 680 participants over a 10.5-year median follow-up, HDL anti-inflammatory capacity induced mRNA expression of vascular cell adhesion molecule 1 (*VCAM1*), which was defined as the capacity of HDL to suppress tumor necrosis factor α (TNFα), was independently associated with a reduced risk of experiencing a CVD event, and it greatly improved risk prediction by the Framingham risk score. Notably, HDL anti-inflammatory capacity did not statistically correlate with HDL-C or high-sensitivity C-reactive protein (hsCRP)– one of the most standardized pro-inflammatory markers used to predict CVD [13]. While the cell-based methods used to assess lipoprotein inflammatory capacity pose barriers to implementation in clinical settings, the significant improvement in CVD risk assessment that these measures provide warrants study into how to adapt similar methods that can be feasibly conducted and scaled-up by clinical laboratories.

### 3.3. Oxidative Activity

The capacity of lipoproteins to mediate pro- and antioxidant effects has additionally been shown to offer insight into CVD risk prediction. While the majority of LDL-focused studies explore the oxidative susceptibility of LDL particles in the presence or pro-oxidants or potential antioxidant therapeutics, studies focused on HDL often explore the antioxidant capacity of the lipoprotein fraction, or the capacity of HDL to suppress the oxidation of target compounds in cell-free biochemical assays [111,112,113]. HDL antioxidant activity is critical in the protection of LDL from oxidation, maintaining HDL particle integrity to facilitate efflux and anti-inflammatory properties, and the overall mitigation of atherosclerotic plaque development. HDL antioxidant capacity assays may be non-specific and can aim to investigate total antioxidant capacity or aim to evaluate the antioxidant activity of specific enzymes known to be carried by HDL, such as PON1/3, based on the substrates included in the assay [14]. Accordingly, Karami et al. [114] demonstrated that patients with metabolic dysfunction-associated steatotic disease (previously classified as non-alcoholic fatty liver disease) who had decreased HDL antioxidant activity had worse cardiometabolic profiles and increased carotid intima–media thickness compared to healthy controls. Reduced HDL antioxidant capacity is additionally observed in CVD patients, in addition to patients at greater risk for CVD, including those with rheumatoid arthritis, chronic kidney disease, and type 2 diabetes [115]. Interestingly, the antioxidant capacity of HDL additionally shows predictive value in conditions beyond CVD and metabolic/inflammatory disorders, including breast cancer [116]. Thus, given the cell-free nature of measurement methods, and the scope of conditions in which HDL antioxidant activity is relevant, implementation of this measure in clinical laboratories may be feasible and strengthen the assessment of risk predictions.

### 3.4. Activity of Proteins Involved in Lipoprotein Metabolism

In addition to measuring the functional properties of lipoproteins, assessing the activity of lipoprotein-associated proteins and enzymes involved in lipoprotein remodeling and metabolism show predictive value for CVD. Some of the most well studied proteins include cholesterol ester transfer protein (CETP), phospholipid transfer protein (PLTP), and lecithin cholesterol acyltransferase (LCAT) [117,118,119]. CETP mediates the exchange of cholesteryl esters from HDL to apoB-containing lipoproteins in exchange for TAG, whereas PLTP mediates the transfer of phospholipids from TAG-rich lipoproteins to HDL [117,120]. LCAT mediates the esterification of cholesterol, predominantly acting on HDL (alpha-LCAT activity) as compared to apoB-containing lipoproteins (beta LCAT activity) [121]. The study of these proteins within the context of CVD risk is supported by cases in which patients present with genetic mutations or variants that the alter function of these proteins [118,122,123]. However, in the general population, measurements of the plasma activity of these proteins can support the assessment of CVD risk. For example, studies have demonstrated that plasma PLTP and CETP activity are positive predictors of CVD events and death [124,125]. Conversely, while low plasma LCAT activity has been reported in patients with CAD or acute myocardial infarction [126,127], the relationship between LCAT activity and CVD risk appears to be more complex [128], with weaker evidence to suggest its implementation in clinical practice for CVD screening in the absence of genetic mutations.

### 3.5. Summary of Dynamic Translational Biomarkers of Lipoprotein Metabolism and Function to Predict CVD Risk

Despite the strong potential for utilizing dynamic measures of lipoprotein metabolism and function in assessing the risk, diagnosis, and recurrence of CVD-related events [72,93], there are barriers to implementing these measures in clinical settings, including an increased need for standardization, reduced cost, and having to potentially modify current cell-based methods to approaches that are more rapid and easily scalable. A summary of lipoprotein function and metabolism biomarkers, including measurement methods, predictive strengths and limitations for CVD risk, and barriers to implementation in clinical practice are highlighted in Table 2. Continued research is needed to explore methods that better optimize these measures for clinical assessment and strengthen therapeutic and preventative strategies that target CVD.

## 4. Omics-Based Measures of Lipoprotein Profiles to Predict CVD Risk

The advancement of omics-based technologies and approaches have expanded the capacity to identify new biomarkers for a broad range of health conditions. However, many omics-based analyses come with a high analytical cost and require advanced instrumentation and technical skills, which limit the capacity to implement in clinical settings. Further, omics-based approaches additionally yield significant amounts of data, and researchers are working toward standardized and validated approaches to analyze and leverage these data in clinically meaningful and realistic ways [20]. As such, the relevance of omics-based data in enhancing CVD predictive scores remains to be further elucidated. Despite these factors, the strengths of including omics-based approaches in research studies may still provide insight into potential mechanisms of lipoprotein regulation and identify new targets or parameters to consider for personalized nutrition therapies [34], and it is expected that in future years, the use of omics will provide more precise diagnostics and will be more utilized in clinical practice as technological approaches become more standardized and cost-effective [133]. The following sections outline current omics-based approaches that have yielded new lipoprotein biomarkers relevant to CVD, with a focus on lipidomics and proteomics, since to date, these techniques have been most relevant to the characterization lipoproteins. It is important to note that omics-based approaches are diverse and complex, and recent omics-focused reviews provide a more detailed overview of plasma- and lipoprotein-specific lipidomic and proteomic applications in biomedical and cardiometabolic disease research [16,60,134]. For the purposes of this review, we provide an overview of plasma and lipoprotein fraction-specific lipidomic and proteomic research and its relevance to predicting and diagnosing CVD, as summarized in Table 3. A discussion of metagenomics and gut microbiome-related metabolites that have relevance to lipoproteins and CVD risk is included in Section 7 below.

### 4.1. Lipidomics

Lipidomics analysis refers to large-scale, comprehensive measures of lipids in a given sample using either targeted or untargeted mass spectrometry-based approaches. Lipidomics can lead to the identification of global patterns of lipid profiles and species, or to the potential identification of new lipid species [153]. In the assessment of CVD risk, lipidomic approaches have been used to characterize lipid profiles in plasma and serum and have identified specific species that are negatively and positively correlated with CVD risk. For example, in a recent comprehensive review of 16 cohort studies, Ding and Rexrode [16] identified phosphatidylcholine species with polyunsaturated fatty acyl chains as negative predictors of CVD outcomes, whereas phosphatidylcholine species containing saturated and monounsaturated fatty acyl chain and specific ceramide species where characterized as being positively associated with CVD risk. However, research has demonstrated that analysis of lipidomic profiles in specific lipoprotein subfractions may offer greater diagnostic and predictive value, as lipoproteins differ in the relative composition of specific lipid species linked to CVD. Further, lipid species that are identified in both HDL and apoB-containing lipoproteins may confer unique effects based on the differing biological and CVD-related functions of each type of lipoprotein [16,154]. In HDL specifically, positive associations with CHD risk are observed in participants with HDL characterized by lower overall phospholipid, sphingomyelin, and PUFA content, yet higher TAG [16,137,155]. Importantly, HDL particles with these atherogenic lipid profiles have also been shown to exhibit decreased cholesterol-accepting capacity in mechanistic cell-based studies [155]. Further, dietary strategies aimed at improving cardiometabolic risk profiles often coincide with improvements in plasma and lipoprotein lipidomic profiles [156,157,158,159]. It is clear that lipidomics approaches offer promising insight, and that the observed biomarkers appear to serve as strong predictors of CVD risk beyond plasma lipids and lipoprotein levels. However, whether lipidomic analysis offers greater diagnostic and prognostic value than traditional biomarkers cannot be determined until the available data become more substantiated with the addition of lipidomics analyses across diverse populations.

### 4.2. Proteomics

Proteomics is defined as the tool that characterizes protein structure, function, protein–protein interactions, and associated protein modifications by utilizing mass spectrometry approaches. Proteomics gives an insight into the perturbation of signaling pathways within tumor cells, has improved the discovery of new therapeutic agents, and is also an indicator of response and duration therapy [160]. Common biomarkers used to identify dyslipidemias measure plasma lipids, but these lack the sensitivity and specificity for early detection [161]. Therefore, more sensitive biomarkers such as those based on proteomics technology are needed to improve early diagnosis [162]. The identification of large numbers of proteins expressed in cells is considered to be key in proteomics since the protein domain is that most affected by disease and also during therapeutic recovery [163]. Du et al. [17] conducted a proteomic analysis in plasma from 30 normal individuals and 30 dyslipidemic patients. Proteomic analysis identified 137 differentially expressed proteins between the healthy individuals and the diseased patients, 60 of which were up-regulated and 77 down-regulated. A detailed analysis showed that most of the proteins, differentially expressed, were located in the extracellular domain, indicating that the extracellular response to dyslipidemia was active. An analysis of protein–protein interactions showed that the differences in patients with dyslipidemia were associated with immunity and inflammation, lipid metabolism, and oxidation. To understand these results, it is important to note that abnormal blood lipids can lead to inflammation [17].

Other studies have identified that people with dyslipidemia have shown altered apolipoprotein patterns in plasma. For example, apoC-I is a protein secreted by fat cells, which is increased in, and predictive of, CVD [164]. ApoAs and apoE are involved in lipid metabolism and participate in the efflux of cholesterol from cells, therefore preventing atherosclerosis [165]. Other apolipoproteins including apoC4, apoF, apoD, apoE, and apo(a) are altered in cases of dyslipidemia, and these alterations interfere with the regulation of immunity and inflammation. Apolipoproteins can inhibit or enhance T cell proliferation, promote the delivery of lipid antigens, and regulate macrophage function [166,167,168,169,170]. Notably, most studies evaluating the association between apolipoproteins and CVD risk utilize targeted protein-specific assays, such as ELISA, rather than global mass spectrometry approaches. Accordingly, the number of studies and strength of evidence linking apolipoproteins to CVD risk is stronger, and measurement of certain apolipoproteins is possible through clinical laboratories.

Similarly to what has been observed in lipidomics analysis, lipoprotein-specific proteomics characterization may offer enhanced insights into CVD risk and lipoprotein functionality [18,171]. While the LDL proteome is reported to be more stable and limited to up to 22 proteins, up to 285 unique proteins have been found in HDL subtractions, with functions ranging across lipid transport and metabolism, pathogen defense, pro- and anti-inflammatory and antioxidant activity, immunoregulation, cell and heparin binding, hemostasis and protease inhibition [60,172,173]. Accordingly, HDL subfractions with increased prevalence of proatherogenic proteins are observed in patients of ischemic stroke and acute coronary syndrome [146,147], in addition to populations at greater risk for CVD, including those with type 1 and 2 diabetes [148,149], chronic kidney disease [18], and autoimmune conditions such as rheumatoid arthritis and psoriasis [150,151]. Preliminary evidence additionally suggests that interventions aimed at improving cardiometabolic risk profiles correspond to improvements in lipoprotein protein composition [152]. Thus, the application of large-scale, global proteomics may provide greater insights into understanding dyslipidemia as well as also forming a framework to better understand the function of lipid-lowering drugs and dietary/lifestyle interventions. However, the number of research studies demonstrating the validity, feasibility, and added utility of incorporating proteomic approaches into clinical assessment needs to be increased to draw solid conclusions regarding this methodology.

## 5. Summary of Lipoprotein Biomarkers to Assess CVD Risk: Implications for Improved Diagnostics and Interventions

As outlined above, various standardized clinical measures and emerging functional and omics-based techniques have been established to characterize lipoproteins within the context of CVD risk. While many of these biomarkers have been shown to be independent predictors of CVD outcomes across research studies, large-scale studies that include these approaches in a comprehensive and comparative manner are lacking. Further, with the goal of leveraging both clinical and emerging biomarkers into clinical settings to improve CVD diagnostics, risk assessment, and design to be more effective in preventative and therapeutic interventions, it is essential to recognize and account for interindividual variability and known determinants of difference in lipoprotein profiles. For example, differences in plasma lipids, the presence of modified and oxidized lipoproteins, activity of proteins involved in lipoprotein remodeling, lipoprotein composition, and parameters of lipoprotein function (e.g., HDL-mediated cholesterol efflux capacity) differ across age groups, and by sex, race/ethnicity, and the presence of pre-existing health conditions and medication use. Accordingly, specific lipoprotein biomarkers serve as stronger vs. weaker predictors of CVD risk across studies and populations, which may correspond to variability in individual responses to preventative and therapeutic treatments [21,22,23,24,25,146,147,152,174]. Thus, in order to improve upon CVD risk assessments and intervention strategies, it is essential to account for an individual’s demographic characteristics known to influence lipoprotein profiles and CVD risk, in addition to known genetic, metagenomic and metabolomic, and lifestyle characteristics that are known determinants of lipoprotein profiles and CVD outcomes [34,35,174]. Therefore, the following sections outline the research and clinical application of these lipoprotein determinants that likely impact the development of precision nutrition recommendations.

## 6. Genetic and Epigenetic Determinants of Lipoprotein Profiles to Predict CVD Risk

Research and clinical case studies have identified numerous gene variants and SNPs that are linked to CVD risk. This includes variations in genes that express apolipoproteins, lipoprotein-associated transporters and receptors, and enzymes involved in lipoprotein metabolism such as PLTP, CETP, and LCAT, among others [118,122,123,175,176,177,178]. These findings have led to the characterization of mechanisms underlying CVD pathophysiology [179], the development of both successful and failed pharmacological therapeutics [180,181], and the identification of diet–gene interactions [7]. Independent of differences in genetic code, increasing evidence demonstrates the importance of considering epigenetic factors in determining CVD risk, including global and gene-specific methylation patterns and the activity of enzymes and regulatory factors involved in DNA methylation, histone modification, and the expression of noncoding RNAs [182]. This review will focus on some of the most well-established genetic factors associated with hypercholesterolemia and hypertriglyceridemia, in addition to emerging epigenetic factors that show diagnostic and predictive potential in CVD.

### 6.1. Apolipoprotein E

ApoE polymorphisms (apoE2, apoE3 and apoE4) have been strongly associated with CVD risk [183]. ApoE3, the parent form, is associated with normal plasma lipids due to apoE recognition by liver receptors (LDL receptor (LDL-R), lipoprotein-related receptor, and heparin sulfate proteoglycan) and timely removal from circulation [184]. In contrast, in apoE2, the substitution of the amino acid arginine in position 158 by cysteine impedes the interaction with the receptors and results in an inability to remove TAG-rich lipoproteins [185]. This polymorphism results in high plasma TAG associated with type-III hyperlipoproteinemia. ApoE4 differs from apoE3 in the substitution of cysteine 112 by arginine. This substitution does not affect the binding to LDL-R; however, it results in an enhanced lipid binding ability that impedes the processing of VLDL in plasma and increases risk of CVD [186]. ApoE4 is also associated with increased risk of Alzheimer’s disease due to its ability to affect several important steps in the amyloid cascade and its impaired ability in mediating β-amyloid clearance through cell surface receptors [187]. Thus, the presence of the E2 allele in 8% of the population and of the E4 allele in 14% of the population predisposes these individuals to increased lipid abnormalities and therefore increased CVD risk. Few studies have additionally investigated the effects of *APOE4* variants on parameters of HDL function, with conflicting findings being reported, which may in part be related to variability in the HDL samples and cell models being studied [188,189,190].

### 6.2. Mutations in Familial Hypercholesterolemia

Familial hypercholesterolemia (FH) is an autosomal dominant disorder that results in elevated LDL-C through mutations in several genes including the *LDLR*, *APOB,* and *PCSK9* [191]. Of these three mutations, those associated with LDL-R are the most common and the ones that present more gene variants. The prevalence of heterozygous FH is about 1 in 220, while the homozygous is prevalent in 1 out of 300,000 patients. Without treatment, heterozygous individuals have a reduced life expectance of 15–30 years, while for homozygous, the life expectance can be 20 years or less and the first heart attack can occur at 2 years of age [192]. Genetic testing is needed for the confirmed diagnosis of FH, and the type of mutation also carries its own risk [193].

In individuals with hypercholesterolemia, LDL-R will be defective in 75% of cases. The type of mutations present in the LDL-R could be a defective receptor or an unfunctional receptor [194]. Mutations are classified according to the function that is impaired: 1. Lack of a functional LDL-R protein often affecting the promoter region. 2. Partial or complete retention of the receptor in the endoplasmic reticulum, thus causing failure for the LDL-R to be transported to the cell membrane. 3. Mutations in the LDL-R that lead to impaired capacity to bind to apo B. 4. Reduced capacity for receptor-mediated endocytosis. 5. Diminished recycling capacity of the receptor [195].

A mutation in *APOB* results in a dysfunctional protein that inhibits LDL particles from binding to LDL-R for cellular uptake and clearance from systemic circulation. This is a less common mutation; however, in a cohort of 825 hypercholesterolemic patients, 12% presented *APOB* variants while the frequency in the general population was about 0.5% [196]. PCSK9 is a relatively newer target for CVD therapies, which functions by degrading the LDL-R. Multiple *PCSK9* gene variants have been associated with a gain-of-function for this protein, resulting in increased degradation of the LDL-R and increases in LDL-C and CVD risk [197], whereas loss-of-function *PCSK9* gene variants are associated with lower plasma LDL-C and CVD risk [198]. Diverse gene variants associated with increased PCSK9 activity have been identified in diverse populations including Norwegian, French, British, Japanese, Chinese, and Turkish [199], whereas pharmacological inhibitors of PCSK9 result in reductions in LDL-C in more than 50% [200]. However, it is important to note that for the management of HF, in addition to lipid-lowering therapy, lifestyle modifications are strongly recommended [201].

It is important to note that FH is additionally associated with alterations in HDL profiles. For example, Ganjali et al. [202] found that the cholesterol efflux capacity of larger HDL2 and smaller HDL3 subfractions was reduced in homozygous FH vs. healthy patients. Differences in HDL composition and function have also been reported between FH, with *PCSK9* gain-of-function FH patients exhibiting reduced anti-inflammatory, antioxidant, anti-thrombotic, and antiapoptotic activity, as well as differences in proteomic, lipidomic, and glycomic composition compared to healthy controls and *LDLR* variant FH [203].

### 6.3. Epigenetic Regulation of Lipoprotein Profiles

Independent of differences in genetic code, increasing evidence demonstrates the importance of considering epigenetic factors in determining CVD risk, including global and gene-specific methylation patterns and the activity of enzymes and regulatory factors involved in DNA methylation, histone modification, and expression of noncoding RNAs, including microRNAs (miRNAs) [182]. Regarding global DNA methylation and CVD risk, inconsistencies have been reported, which is likely due to the complexity and specificity of the tissues and genomics regions being studied. For example, the hypermethylation of repetitive ALU elements in leukocytes is associated with increased CVD risk [204]. Conversely, global DNA has been found to be hypomethylated in atherosclerotic tissue, yet hypermethylated in the promoted regions of atherosclerotic genes [205,206,207]. By conducting epigenome-wide analysis from the Registre Gironi del Cor (REGICOR) study, Sayols-Baixeras et al. [208] identified gene-specific methylation sites associated with both cholesterol efflux capacity and HDL inflammatory indices. In addition to DNA methylation, patterns of histone modifications and the activity of histone-modifying enzymes such as histone methyltransferases, acetyltransferases, and deacetylases, including nicotinamide adenine dinucleotide (NAD+)-dependent sirtuins, have additionally been linked to dyslipidemias and CVD risk [209]. Noncoding RNAs, including specific microRNAs, long noncoding RNAs, small interference RNAs, and circular RNAs, have been associated with both increased and decreased risk of CVD, and are known to be involved in the regulation of lipoprotein biogenesis, metabolism, and hepatic and macrophage uptake and efflux. Accordingly, noncoding RNAs have been proposed as novel diagnostic and therapeutic targets for CVD. The role of noncoding RNAs in lipoprotein metabolism and CVD risk has been reviewed in detail by others [210,211,212]. Taken together, epigenetics may be an important consideration for the implementation of precision nutrition recommendations aimed at reducing CVD risk [213].

## 7. Metagenomic (Gut Microbiome) and Related Metabolomic Determinants of Lipoprotein Profiles and CVD Risk

The gut microbiota has a very important role in nutrient metabolism through its various functions, including defense against pathogens, vitamin synthesis, production of short-chain fatty acids (SCFA), activation of the immune system, and maintenance of intestinal barrier function [214]. The gut microbiota has a strong symbiotic relationship with the host, and it can affect human physiology and pathology [215]. There are specific metabolites, including SCFA and trimethylamine oxide (TMAO), that have been identified to have a strong association with dyslipidemia. Changes in microbiota structure can affect the occurrence and development of dyslipidemia. In addition, dyslipidemia further aggravates dysbiosis [216].

Dietary fiber is fermented by the gut microbiota and there is a production of SCFA, including acetic, propionic, and butyric acid. It has been shown that increasing the abundance of bacteria that produce these fatty acids results in significant reductions in plasma lipids [217] and improvement in dyslipidemia [218]. Firmicutes and Bacteroides are the main phyla that can influence concentrations of plasma cholesterol, TAG, and HDL-C [219]. Therefore, knowledge regarding the existence of SCFA-producing bacteria in the host can provide information on their protective effects against dyslipidemia.

Choline and carnitine present in foods can also be transformed by the gut microbiota into trimethyl amine (TMA). This metabolite then travels to the liver where it can be oxidized into TMAO by the action of the enzyme flavin monoxoxygenase-3 [220]. Several studies have demonstrated that plasma TMAO concentrations are correlated with cardiovascular disease. Plasma TMAO levels are higher in CHD patients compared to healthy subjects [221]. TMAO has been observed to reduce the expression of cholesterol 7-alpha hydroxylase [222] and to promote uptake of cholesterol in macrophages by the increasing the expression of scavenger receptors [223]. Increased concentrations of plasma TMAO have also been shown to have a negative impact on RCT and decrease plasma HDL-C concentrations [224]. Therefore, higher chronic concentrations of TMAO, not increased transitory concentrations due to diet, are a recognized biomarker for dyslipidemia and heart disease [220].

Importantly, increasing evidence suggests gut microbiome profiles are critical determinants of the diet’s effects on a wide range of biological processes, and that differences in baseline gut microbiome profiles are in part attributable to interindividual variability in responses to dietary intervention [225]. Thus, accounting for baseline gut microbiome profiles is likely to be essential in the design of precision nutrition studies.

## 8. Lifestyle Determinants

Lifestyle plays a strong and unique role in the development of dyslipidemias that lead to heart disease [226]. Some of the lipoprotein biomarkers discussed in this review are related to specific metabolic disturbances that are primarily caused by unhealthy dietary habits, use of alcohol and smoking, obesity, stress, and lack of physical activity [28]. These factors may not only be targets for precision nutrition recommendations but considerations for the design of research studies aimed at evaluating whether these factors influence individualized responses to dietary intervention.

### 8.1. Diet

There are dietary habits that are directly associated with major dyslipidemias that increase the risk for heart disease [227]. The intake of high-carbohydrate diets leads to increases in plasma TAG and lowers HDL-C, as well as increasing inflammatory compounds [228]. High carbohydrate diets have also been correlated with insulin resistance and type 2 diabetes that eventually will lead to CVD [229]. A high intake of simple sugars has also been related to metabolic syndrome [230], which in turn doubles the risk of CVD [231]. Other nutrients associated with an increased risk of CVD are trans fatty acids [232] because they not only increase LDL-C but also reduce HDL-C, leading to a more atherogenic lipoprotein profile [65].

Saturated fatty acids (SFAs) were considered the most relevant nutrient associated with increases in plasma cholesterol [233]. However, the whole premise of SFAs’ involvement in CVD has been questioned [234,235]. Some of the evidence comes from the Nurses’ Health study that found, after 20 years of follow-up, a flat dose–response relationship was observed between SFA and CHD risk [236]. Also, saturated fat appears to increase HDL-C and the size of the LDL particles, which are less atherogenic than small dense LDLs [38]. Small LDL particles become oxidized more easily and are easily deposited in the arterial wall, initiating the process of atherosclerosis [237].

The lack of a correlation between dietary cholesterol and increases in the biomarkers for CHD risk has also been shown in numerous clinical studies [238]. Further, clinical interventions with eggs, a major source of dietary cholesterol, have shown a consistent maintenance of the LDL-C/HDL-C ratio [239,240] and increases in large LDL, the less atherogenic particle [241]. Studies have also demonstrated that eggs increase the functionality of the HDL particle by more effectively removing cholesterol from macrophages ex vivo, and the particles have been associated with changes in HDL lipid and associated protein composition [97,157]. Based on some of these studies, the USDA dietary guidelines removed upper limits for dietary cholesterol in 2015, further documenting that there is no correlation between dietary cholesterol and risk for CVD [242]. The current 2020 USDA dietary guidelines have not changed this concept [243].

There are also numerous compounds that have been shown to protect against CVD, including carotenoids [244,245], polyphenols [246,247], and certain dietary fatty acids [248]. Therefore, a diet rich in fruits and vegetables, omega (ω)-3 fatty acids, and antioxidant vitamins and minerals can be considered protective against CVD. Further some dietary approaches have been found useful to improve the functionality of HDL. For example, monounsaturated fatty acids increase PON1 activity, which is associated with greater HDL antioxidant capacity, while oxidized lipids reduce its activity [249]. Also, fruits and vegetables that are high in Vitamin E and other antioxidants have demonstrated a protective effect against LDL oxidation [250]. Taken together, it is clear that a wide variety of dietary compounds influence lipoprotein metabolism and pathways involved in CVD pathophysiology and should thus be considered as targets or potential confounders in precision nutrition research and recommendations.

### 8.2. Smoking and Alcohol

In addition to its oxidative effects that lead to the formation of reactive oxygen species (ROS) and increased risk for heart disease [251], smoking has been demonstrated to increase serum concentrations of oxidized LDL antibodies as well as decreased concentrations of plasma carotenoids [252]. Smoking has also been associated with low HDL-C, while smoking cessation increases this lipoprotein [253]. The Interlipid study, comprising 789 Japanese (45–65 y) using multiple variate linear regression, evaluated correlations between smoking, alcohol use, and body mass index. Higher smoking was associated with a lower number of HDL particles and lower concentrations of HDL-C [254].

Although alcohol in moderation has been associated with protection against CVD [255], it has been known for decades that a high intake of alcohol is correlated with disruption of lipoprotein metabolism, fatty liver, hepatic steatosis, and cirrhosis [256]. In addition, high alcohol intake has been correlated with low PON activity, and lower levels of both HDL-C and apoA-I concentrations [257]. Thus, restriction of alcohol and cessation of tobacco use are highly recommended to improve dyslipidemias and thus prevent CVD [258].

### 8.3. Obesity

Obesity has been correlated with major dyslipidemias including increases in LDL-C and plasma TAG and reductions in HDL [259], as well as the presence of small LDL [260]. A reversal of these conditions has been observed even with moderate weight losses in numerous clinical studies [261,262]. Interestingly, as little as 5% weight loss is beneficial for dyslipidemia and further improvement is observed with greater increases in weight loss from 11 to 16% [262]. The adipose tissue is a main site for the deposition of cholesterol and triglycerides [263]. Obesity leads to a higher deposition of these lipids, which results in insulin resistance and the induction of pro-inflammatory cytokines [264]. This, in turn, leads to the release of more fatty acids in circulation and their uptake by the liver. The liver will then increase the production of VLDL, leading to increases in both plasma triglycerides and cholesterol, and subsequent hyperlipidemias [265]. Obesity is considered a major contributor to CVD risk since it has been established to be associated with atherosclerosis, heart failure, arrythmias, and atrial fibrillation [266].

### 8.4. Sedentary Lifestyle and Physical Activity

Sedentary lifestyle and decreased level of engagement in physical activity have been associated adverse lipoprotein profiles and increased CVD risk [267,268,269]. In an observational study from the Observation of Cardiovascular Risk Factors in Luxembourg (ORISCAV-LUX) cohort, sedentary lifestyle measures were positively associated with blood concentrations of total cholesterol, LDL-C, and TAG in healthy-weight adult participants, whereas higher HDL-C concentrations were observed in participants who engaged in less screen time and greater intense physical activity [270]. Associations between dyslipidemia and physical activity have additionally been observed in children [271]. Exercise interventions—particularly aerobic exercise—have also been shown to improve measures of HDL function, including efflux capacity, and antioxidant and anti-inflammatory activity; however, the results are not always consistent due to variability in study populations and exercise protocols [272].

### 8.5. Stress

Stress is an increasingly well-characterized risk factor for CVD. Importantly, various forms of stress have been linked to dyslipidemias, including acute and chronic stress, as well as stress stemming from post-traumatic stress disorder, anxiety, and depression [273,274,275,276]. These associations are not only attributable to the practice of less healthy lifestyle patterns, including the consumption of nutrient-poor, atherogenic diets, limited physical activity, and poor sleep hygiene, but also metabolic dysfunction promoted by elevated levels of stress hormones, such as cortisol [277]. Accordingly, interventions that promote stress management and physical activity, such yoga, have been shown to improve plasma lipid profiles [278]. Further, higher levels of stress have been associated with lower adherence to healthful diet prescriptions [279]. Thus, stress may be an important consideration in designing precision nutrition interventions.

## 9. Conclusions, Limitations, and Considerations

As outlined in this review, there is a range of important factors to consider when designing precision nutrition interventions aimed at targeting lipoprotein metabolism to reduce CVD risk for results and to ultimately contribute to our understanding of diet–disease associations that can be translated into clinical practice. It is essential to continue to study and explore the standard and emerging lipoprotein biomarkers in comprehensive and comparative studies to determine which lipoprotein profiles provide the greatest diagnostic and predictive value for CVD risk, and whether the diagnostic and predictive value of specific biomarkers differs across populations or patient groups. The rapidly increasing range of techniques used for the identification of small molecules and proteins or lipids associated with increased risk for chronic disease will become more useful as new biomarkers capable of providing a more accurate diagnosis are being discovered and validated. Currently, the most traditional methods of measuring plasma lipids and lipoproteins continue to be used as biomarkers to determine CVD risk due to their lost cost, standardization, and availability across clinical laboratories, coverage by medical insurance, and validity from many large-scale trials; however, as previously described, these new methodologies provide additional information that will likely lead to a more precise diagnosis.

In order to effectively leverage emerging biomarkers for clinical CVD risk prediction and diagnosis, it is important to recognize and address current limitations and barriers in their analysis and validation for addressing CVD outcomes. First, it is important to note that many biomarker-CVD risk associations come from observational studies rather than clinical trials. Observational studies can have inherent bias that can influence results, such as selection bias in participant grouping or data selected for analysis, information bias such as errors in measurement or recall of self-reported data by participants, the inclusion or exclusion of confounders in statistical models, and researcher bias in interpretation [280]. Further, the variability in methods for assessing biomarkers such as proteomic profiles and lipoprotein function (e.g., HDL inflammatory capacity) is high, which could lead to discrepancies in CVD risk associations. Thus, the need to study emerging lipoprotein biomarkers by utilizing standardized methods in large-scale clinical trials is warranted.

## 10. Future Perspectives and Recommendations for Precision Nutrition Interventions That Target Lipoprotein Profiles and CVD Risk

The field of precision nutrition has approached a novel, exciting, and pivotal time for advancing research. However, the mechanisms by which to implement and conduct precision nutrition research, in general, is still being developed and defined. In this review, we highlight the known factors that are important to consider as primary and secondary outcomes, including clinical and emerging lipoprotein biomarkers, covariates, definers of participant subgroups, and post hoc analyses, as well as for the study of lipoprotein markers in CVD and precision nutrition research. Accordingly, it will be essential to consider all the determinants of lipoprotein profiles, including genetic, epigenetic, metagenomic, and lifestyle factors in the design of precision nutrition research studies. In addition, the stratification of participants from diverse populations into control vs. experimental groups will be necessary in order to determine interindividual variability in lipoprotein and cardiometabolic responses to diet. Researchers will need to develop standardized, validated, and accessible approaches to data handling and analysis to appropriate capture patterns and clinically significant findings in precision nutrition studies that encompass large amounts of complex data [34,35]. Together, these approaches will provide greater insight into the potential impact of precision nutrition approaches in targeting lipoprotein profiles and CVD risk to improve clinical outcomes.

## Figures and Tables

**Table 1:** Traditional clinical lipoprotein biomarkers and their interpretation and clinical applicability for identifying dyslipidemia and risk for heart disease.

Biomarker	Method(s) of Measurement	Clinical Implementation	Clinical Interpretation and Relevance to CVD	Limitations/Complications in Clinical Interpretation and Relevance to CVD	References
Plasma lipid panels					
Total cholesterol (TC)	Colorimetric method	Routinely used in clinical settings Standardized methodology across clinical and research labs Rapid results Low cost Utilized for CVD risk assessment in the general population and populations at high-risk for CVD Common universal coverages by medical insurance providers Commonly used for primary risk assessment and endpoints in CVD public health initiatives and therapeutic trials	High TC is associated with ↑ CVD risk	Does not reflect the ratio of LDL-C/HDL-C or atherogenicity of lipoprotein particles.	[68,69]
Plasma LDL-C	Colorimetric method/calculation with Friedewald equation		High LDL-C is associated with ↑ CVD risk	Individuals with low LDL-C experience CVD events. Accuracy of LDL-C calculations by the Friedewald equation is negatively impacted by high TAG.	[70]
Plasma HDL-C	Colorimetric method after precipitation of apoB containing lipoproteins		Low HDL-C has historically been associated with ↑ CVD risk	More recent research shows a U-shaped relationship with low and high HDL-C increasing CVD risk. Therapeutics aimed at raising HDL-C do not ↓ CVD risk. HDL function (e.g., efflux capacity) shows greater strength in CVD prediction.	[39,41,71,72,73]
LDL-C/HDL-C ratio	Calculation		High LDL-C/HDL-C ratio is associated with ↑ CVD risk and may be a better indicator of CAD severity vs. LDL-C and HDL-C alone	Cutoffs for optimal ratios vary across studies and populations. Accuracy of ratio can be negatively impacted by TAG if LDL-C is calculated via the Friedewald equation. Recent study reports a U-shaped relationship with low and high LDL-C/HDL-C ratio increasing all-cause mortality risk.	[43,74,75,76]
Plasma TAG	Colorimetric method		High TAG is associated with ↑ CVD risk	Influenced by recent food intake and relatively more variable throughout the day and day-to-day compared to other lipids.	[46,77,78]
**Apolipoproteins**					
ApoA-I	Immunoassays (ELISA, multiplex assays), NMR, proteomics	Available for measure by clinical laboratories Higher cost compared to plasma lipids, but increased accessibility and feasibility by clinical laboratories has made measures more cost-effective May not be covered by medical insurance for preventative screening	Correlates with number of HDL particles Inversely associated with CVD risk	Does not capture functionalaity of HDL (e.g., efflux and inflamamtory capacity).	[79,80]
ApoB	Immunoassays (ELISA, multiplex assays), NMR, proteomics		Biomarker for the number of TAG- and cholesterol-rich lipoprotein (VLDL, LDL) particles Positively associated with CVD risk Shows greater CVD predictive strength compared to other lipids in some studies	May not fully capture atherogenicty of apoB-containing particles (e.g., particle size, oxidation state).	[81,82,83]
ApoB/ApoA-I ratio	Ratio calculation		Reflects the ratio of VLDL/IDL/LDL: HDL particles Shown to better predict CVD vs. plasma lipids and other lipoprotein markers in some studies	Does not capture HDL functionality or atherogenicity of apoB-containing lipoprotein particles. Data are inconsistent on whether apoB/apoA-I ratios provide greater diagnostic and predictive potential over other more feasible and cost-effective lipid measures.	[44,80,84,85,86]
**Plasma lipoprotein subfractions by particle size and number**					
VLDL size and particle number	NMR, ion mobility, gel electrophoresis	Available for measure by select clinical laboratories Higher cost and time for analysis than plasma lipid measures May not be covered by medical insurance for preventative screening	High concentrations of large VLDL indicative of hypertriglyceridemia and ↑ CVD risk	Small VLDL and VLDL-cholesterol may serve as better indicators of CVD risk during statin therapy.	[52,87]
LDL size and particle number	NMR, ion mobility, gel electrophoresis		LDL particle size and number is inversely associated with CVD risk LDL particle number shown to be a better predictor compared to other lipid measures in some studies	Inconsistent findings are reported on whether LDL particle size and number are greater predictor of CVD events compared to other lipid measures.	[38,88]
HDL size and particle number	NMR, ion mobility, gel electrophoresis		HDL particle size and number is inversely associated with CVD risk	Small HDL often shows increased antioxidant capacity in functional experiments.	[14,57]
**Modified lipoprotein biomarkers**					
Lipoprotein (a)	Apo(a)-specific immunooassay	Available for measure by select clinical laboratories Higher cost and time for analysis than plasma lipid measures May not be covered by medical insurance for preventative screening	Concentration > 30 mg/dL is associated with increased risk for CVD	Lp(a) has a very strong genetic component, with conflicting associations with diet and lifestyle interventions.	[61,89,90]
Oxidized LDL	ApoB-specific immunooassay		Higher concentrations are observed in populations with or at high risk for heart CVD	The strength of eviednce for oxidized LDL to serve as an independent predictor of CVD is inconsistent and relatively weaker comapred to other clinical lipid measures.	[15,62,91,92]

**Abbreviations and symbols**: ↓: decreased; ↑: increased; Apo: apolipoprotein; CVD: cardiovascular disease; ELISA: enzyme-linked immunosorbent assay; NMR: nuclear magnetic resonance; TAG: triacylglycerol.

**Table 2:** Dynamic and translational biomarkers of lipoprotein function and metabolism and their interpretation and clinical applicability for identifying dyslipidemia and CVD risk.

Biomarker	Method(s) of Measurement	Barriers to Clinical Implementation	Interpretation and Relevance to CVD	Limitations/Complications in Interpretation and Relevance to CVD	References
**Lipoprotein-cell cholesterol flux**					
LDL and apoB-containing lipoprotein uptake	Fluorescence-based or radiolabeled ex vivo cell culture assays	Need for standardization of methods and cell-free alternative approaches Need for methods that are more rapid, scalable, and cost-effective	Predominantly used for mechanistic, basic science studies Used to evaluate uptake of LDL from familial hypercholesterolemia patients vs. controls	Further research is warranted to evaluate whether these measures could offer clinical insight in the assessment of an individual’s CVD risk.	[98,99]
HDL/serum cholesterol-accepting capacity	Fluorescence-based or radiolabeled ex vivo cell culture assays		Inversely associated with CAD severity and CVD risk Shows greater diagnostic potential than HDL-C	Differences in cell models and samples (e.g., HDL vs. whole serum) may lead to greater variability in results across studies.	[72,73]
**Lipoprotein inflammatory activity**					
LDL inflammatory capacity	Ex vivo cell culture-based methods with pro-inflammatory stimuli and assessment of inflammatory cellular and cytokine secretion measures Cell-free colorimetric assays	Need for standardization of methods and cell-free alternative approaches Need for methods that are more rapid, scalable, and cost-effective	Oxidized LDL promotes shift from anti-inflammatory M2 macrophages to pro-inflammatory M1 phenotypes and ↑ foam cell formation LDL from high CVD risk patients have ↑ pro-inflammatory activity	For cell-based assays, differences in cell models (e.g., macrophages, vascular endothelial cells) may lead to greater variability in results across studies. Differences in outcome measures of inflammation (e.g., cytokine secretion in media, cellular gene expression of inflammatory signaling, monocyte chemoattractant activity in coculture) may lead to greater variability in results across studies. Inclusion of this measure in additional large-scale studies that compare its predictive potential for CVD risk relative to other standard lipid measures is warranted.	[109,110]
HDL inflammatory capacity			HDL from patients with high CVD risk have ↑ anti-inflammatory activity compared to LDL Serves as a better indicator of improvements in cardiometabolic profiles in CHD patients Independently associated with reduced risk of experiencing a CVD event, and greatly improves risk prediction by the Framingham risk score.		[13,93,110]
**Oxidative activity**					
LDL oxidizability	Cell-free colorimetric or fluorometric assays	Need for standardization and validation methods—particularly cell-free methods—which are likely to be more rapid, scalable, and cost-effective compared to cell-based methods	Oxidative susceptibility of LDL particles has mainly been studied in mechanistic studies in the presence or prooxidants or potential antioxidant therapeutics	Inclusion of this measure in large-scale studies that compare its predictive potential for CVD risk relative to other standard lipid measures is warranted.	[111,112]
HDL antioxidant activity	Ex vivo cell culture-based methods with pro-oxidant stimuli and assessment of cellular oxidative stress markers Capacity to limit LDL oxidation Cell-free colorimetric or fluorometric assays Activity of HDL-associated antioxidant enzymes (e.g., PON 1/3 activity)		↓ in CVD patients and populations at high risk for CVD Inversely associated with carotid intima-media thickness in patients at high risk for CVD	Inclusion of this measure in large-scale studies that compare its predictive potential for CVD risk relative to other standard lipid measures is warranted.	[113,114,115,129]
**Activity of proteins involved in lipoprotein metabolism**					
Plasma CETP activity	Fluorometric methods	Methods are relatively more standardized, scalable, and cost-effective compared to other dynamic measures of lipoprotein metabolism and function More research is needed to validate biomarkers in clinical settings	High activity is a predictor of ↑ CVD risk	CVD therapeutics targeting CETP have had conflicting and off-target effects, with more recent promise.	[125,130,131]
Plasma LCAT Activity	Fluorometric methods		Low activity reported in patients with CAD or acute myocardial infarction	Relationship between LCAT activity and CVD risk is more complex. Weaker evidence to suggest its implementation in clinical practice for CVD screening in the absence of genetic mutations.	[126,127,128]
Plasma PLTP activity	Fluorometric methods		High activity is a predictor of ↑ CVD risk	Conflicting studies have reported low activity being associated with the presence of peripheral artery disease.	[124,132]

**Abbreviations and symbols**: ↓: decreased; ↑: increased; apo: apolipoprotein; CAD: coronary artery disease; CVD: cardiovascular disease; CETP: cholesterol ester protein; LCAT: lecithin–cholesterol acyl transferase; PLTP: phospholipid transport protein; RCT: reverse cholesterol transport; TAG: triacylglycerol.

**Table 3:** Summary of omics-based lipoprotein biomarkers and their interpretation and clinical applicability for identifying dyslipidemia and risk for CVD.

Biomarker	Method(s) of Measurement	Barriers to Clinical Implementation	Interpretation and Relevance to CVD	Limitations/Complications in Interpretation and Relevance to CVD	References
**Lipidomic biomarkers**					
Plasma lipidome	Targeted/untargeted LC-MS, shotgun lipidomics, NMR-based lipidomics	Lack of standardized methodology and lipoprotein isolation approaches Services not available in clinical laboratories High cost of instrumentation and sample analysis Processing and statistical analysis of large data obtained through lipidomics approaches are complex and variable Utilizing targeted approaches only may miss important lipid-CVD associations More research in large-scale clinical studies is needed to validate the additive predictive value of including these measures	PC species with PUFA acyl chains are negative predictors of CVD PC and ceramide species with SFA and MUFA fatty acyl chains are positive predictors of CVD	Does not account for the distribution of specific lipid species across liporpotein fractions, which can alter their assocition with CVD outcomes.	[16]
Non-HDL/LDL lipidome	Isolation of non-HDL fractions from plasma, targeted/untargeted LC-MS, shotgun lipidomics, NMR-based lipidomics		↑ ceramide in aortic lesion LDL in animal models PC, UFA, SFA, # of FA, and cholesterol are predictors of CHD	Multiple studies evaluated non-HDL lipid compositon rather than LDL-specific lipid composition in relation to CVD.	[135,136]
HDL lipidome	Isolation of HDL fractions from plasma. Targeted/untargeted LC-MS, shotgun lipidomics, NMR-based lipidomics		↑ HDL-TAG and -PE associated with CHD/CVD ↓ HDL-PL, -SM, -PI, -PUFA, PC-derived plasmalogens is associated with CHD/CVD	Inconsistencies reported across studies, with specific lipid species with a given class (e.g., PC and SM) having positive and negative associations with CVD-related measures.	[16,137,138,139,140]
**Proteomic biomarkers**					
Global plasma proteome	MS, aptamer, and immunoaffinity-based approaches	Lack of standardized methodology Services not available in clinical laboratories Relatively higher cost of instrumentation and sample analysis compared to current clinical lipid measures Utilizing targeted approaches only may miss important protein-CVD associations More research in large-scale clinical studies is needed to validate the additive predictive value of including these measures	Differential expression of proteins involved in lipid metabolism/transport, immune regulation, and inflammation in dyslipidmic and CHD patients vs. healthy controls	Does not account for the distribution of specific proteins across liporpotein fractions, which can alter their assocition with CVD outcomes.	[17,141]
Global LDL proteome	Isolation of LDL fractions from serum; targeted/untargeted MS-based approaches		Led to identification of new proteins carried by LDL Characterized distinct proteomic profiles of LDL vs. Lp(a) Increased LDL-SAA in altered apolipoprotein profiles in patients with or at higher risk for CVD	Few studies have conducted untargeted proteomic analysis of LDL/non-HDL subfractions. The capacity of comprehensive LDL proteomic profiles to predict CVD outcomes warrants further investigation in large-scale clinical trials.	[142,143,144,145]
Global HDL proteome	Isolation of HDL fractions from serum; targeted/untargeted MS-based approaches		Differential expression of proteins involved in lipid metabolism/transport, antioxidant activity, immune regulation, and inflammation in patients with or at higher risk for CVD vs. healthy controls, and can be improved with lifestyle intervention	The HDL proteins identified across studies can vary significantly, which may be due to differences in HDL isolation and analysis methods. The capacity of comprehensive HDL proteomic profiles to predict CVD outcomes warrants further investigation in large-scale clinical trials.	[18,146,147,148,149,150,151,152]

Abbreviations and symbols: ↓: decreased; ↑: increased; apo: apolipoprotein; CHD: coronary heart disease; CVD: cardiovascular disease; CETP: cholesterol ester protein; FA: fatty acids; LCAT: lecithin–cholesterol acyl transferase; LC-MS: liquid chromatography–mass spectrometry; MUFA: monounsaturated fatty acid; NMR: nuclear magnetic resonance; PC: phosphatidylcholine; PI: phosphatidylinositol; PL: phospholipid; PLTP: phospholipid transport protein; PUFA: polyunsaturated fatty acid; SAA: serum amyloid A; SFA: saturated fatty acid; SM: sphingomyelin; TAG: triacylglycerol: UFA: unsaturated fatty acids.

## References

[1] Allen J.P. (2005). The Art of Medicine in Ancient Egypt.

[2] Booth J. (1977). A short history of blood pressure measurement. Proc. R. Soc. Med..

[3] Mattes W.B., Goodsaid F. (2018). Regulatory landscapes for biomarkers and diagnostic tests: Qualification, approval, and role in clinical practice. Experim. Biol. Med..

[4] Rouse R., Kruhlak N., Weaver J., Burkhart K., Patel V., Strauss D.G. (2018). Translating New Science Into the Drug Review Process: The US FDA’s Division of Applied Regulatory Science. Ther. Innov. Regul. Sci..

[5] Bossuyt P.M., Reitsma J.B., Bruns D.E., Gatsonis C.A., Glasziou P.P., Irwig L.M., Moher D., Rennie D., de Vet H.C., Lijmer J.G. (2003). The STARD statement for reporting studies of diagnostic accuracy: Explanation and elaboration. Annals. Int. Med..

[6] National Center for Health Statistics (2023). Multiple Cause of Death 2018–2022 on CDC WONDER Database.

[7] Martin S.S., Aday A.W., Almarzooq Z.I., Anderson C.A.M., Arora P., Avery C.L., Baker-Smith C.M., Barone Gibbs B., Beaton A.Z., Boehme A.K. (2024). 2024 Heart Disease and Stroke Statistics: A Report of US and Global Data From the American Heart Association. Circulation.

[8] Andersen C.J., Murphy K.E., Fernandez M.L. (2016). Impact of Obesity and Metabolic Syndrome on Immunity. Adv. Nutr..

[9] Andersen C.J., Vance T.M. (2019). Gender Dictates the Relationship between Serum Lipids and Leukocyte Counts in the National Health and Nutrition Examination Survey 1999(-)2004. J. Clin. Med..

[10] Karaagac Y. (2023). Trimethylamine N-Oxide as a Potential Biomarker for Cardiovascular Disease: Its Association with Dietary Sources of Trimethylamine N-Oxide and Microbiota. Eurasian. J. Med..

[11] Pearson T.A., Mensah G.A., Alexander R.W., Anderson J.L., Cannon R.O., Criqui M., Fadl Y.Y., Fortmann S.P., Hong Y., Myers G.L. (2003). Markers of inflammation and cardiovascular disease: Application to clinical and public health practice: A statement for healthcare professionals from the Centers for Disease Control and Prevention and the American Heart Association. Circulation.

[12] Rohatgi A., Khera A., Berry J.D., Givens E.G., Ayers C.R., Wedin K.E., Neeland I.J., Yuhanna I.S., Rader D.R., de Lemos J.A. (2014). HDL cholesterol efflux capacity and incident cardiovascular events. N. Engl. J. Med..

[13] Jia C., Anderson J.L.C., Gruppen E.G., Lei Y., Bakker S.J.L., Dullaart R.P.F., Tietge U.J.F. (2021). High-Density Lipoprotein Anti-Inflammatory Capacity and Incident Cardiovascular Events. Circulation.

[14] Brites F., Martin M., Guillas I., Kontush A. (2017). Antioxidative activity of high-density lipoprotein (HDL): Mechanistic insights into potential clinical benefit. BBA Clin..

[15] Hong C.G., Florida E., Li H., Parel P.M., Mehta N.N., Sorokin A.V. (2022). Oxidized low-density lipoprotein associates with cardiovascular disease by a vicious cycle of atherosclerosis and inflammation: A systematic review and meta-analysis. Front. Cardiovasc. Med..

[16] Ding M., Rexrode K.M. (2020). A Review of Lipidomics of Cardiovascular Disease Highlights the Importance of Isolating Lipoproteins. Metabolites.

[17] Du H., Rao Y., Liu R., Deng K., Guan Y., Luo D., Mao Q., Yu J., Bo T., Fan Z. (2021). Proteomics and metabolomics analyses reveal the full spectrum of inflammatory and lipid metabolic abnormalities in dyslipidemia. Biomed. Chromatogr..

[18] Shao B., Mathew A.V., Thornock C., Pennathur S., Michigan Kidney Translational Core C.I.G. (2021). Altered HDL proteome predicts incident CVD in chronic kidney disease patients. J. Lipid. Res..

[19] Hoogeveen R.M., Pereira J.P.B., Nurmohamed N.S., Zampoleri V., Bom M.J., Baragetti A., Boekholdt S.M., Knaapen P., Khaw K.T., Wareham N.J. (2020). Improved cardiovascular risk prediction using targeted plasma proteomics in primary prevention. Eur. Heart. J..

[20] Mohr A.E., Ortega-Santos C.P., Whisner C.M., Klein-Seetharaman J., Jasbi P. (2024). Navigating Challenges and Opportunities in Multi-Omics Integration for Personalized Healthcare. Biomedicines.

[21] Simony S.B., Mortensen M.B., Langsted A., Afzal S., Kamstrup P.R., Nordestgaard B.G. (2022). Sex differences of lipoprotein(a) levels and associated risk of morbidity and mortality by age: The Copenhagen General Population Study. Atherosclerosis.

[22] Appelman Y., van Rijn B.B., Ten Haaf M.E., Boersma E., Peters S.A. (2015). Sex differences in cardiovascular risk factors and disease prevention. Atherosclerosis.

[23] Wei Y., Qi B., Xu J., Zhou G., Chen S., Ouyang P., Liu S. (2014). Age- and sex-related difference in lipid profiles of patients hospitalized with acute myocardial infarction in East China. J. Clin. Lipidol..

[24] Zakai N.A., Minnier J., Safford M.M., Koh I., Irvin M.R., Fazio S., Cushman M., Howard V.J., Pamir N. (2022). Race-Dependent Association of High-Density Lipoprotein Cholesterol Levels With Incident Coronary Artery Disease. J. Am. Coll. Cardiol..

[25] Willey J.Z., Rodriguez C.J., Carlino R.F., Moon Y.P., Paik M.C., Boden-Albala B., Sacco R.L., DiTullio M.R., Homma S., Elkind M.S. (2011). Race-ethnic differences in the association between lipid profile components and risk of myocardial infarction: The Northern Manhattan Study. Am. Heart. J..

[26] Bea A.M., Larrea-Sebal A., Marco-Benedi V., Uribe K.B., Galicia-Garcia U., Lamiquiz-Moneo I., Laclaustra M., Moreno-Franco B., Fernandez-Corredoira P., Olmos S. (2023). Contribution of APOE Genetic Variants to Dyslipidemia. Arter. Thromb. Vasc. Biol..

[27] Lee S.H. (2021). Role of Genetics in Preventive Cardiology: Focused on Dyslipidemia. Korean. Circ. J..

[28] Ghodeshwar G.K., Dube A., Khobragade D. (2023). Impact of Lifestyle Modifications on Cardiovascular Health: A Narrative Review. Cureus.

[29] Zaher A., Elsaygh J., Peterson S.J., Weisberg I.S., Parikh M.A., Frishman W.H. (2024). The Interplay of Microbiome Dysbiosis and Cardiovascular Disease. Cardiol. Rev..

[30] Lu L., Jing W., Qian W., Fan L., Cheng J. (2024). Association between dietary patterns and cardiovascular diseases: A review. Curr. Probl. Cardiol..

[31] Lichtenstein A.H., Appel L.J., Vadiveloo M., Hu F.B., Kris-Etherton P.M., Rebholz C.M., Sacks F.M., Thorndike A.N., Van Horn L., Wylie-Rosett J. (2021). 2021 Dietary Guidance to Improve Cardiovascular Health: A Scientific Statement From the American Heart Association. Circulation.

[32] Dynka D., Kowalcze K., Charuta A., Paziewska A. (2023). The Ketogenic Diet and Cardiovascular Diseases. Nutrients.

[33] Feingold K.R., Feingold K.R., Anawalt B., Blackman M.R., Boyce A., Chrousos G., Corpas E., de Herder W.W., Dhatariya K., Dungan K., Hofland J. (2000). The Effect of Diet on Cardiovascular Disease and Lipid and Lipoprotein Levels. Endotext.

[34] Hong B.V., Agus J.K., Tang X., Zheng J.J., Romo E.Z., Lei S., Zivkovic A.M. (2023). Precision Nutrition and Cardiovascular Disease Risk Reduction: The Promise of High-Density Lipoproteins. Curr. Atheroscler. Rep..

[35] Callahan A.E., National Academies of Sciences, Engineering, and Medicine, Health and Medicine Division, Food and Nutrition Board, Food Forum (2021). Challenges and Opportunities for Precision and Personalized Nutrition: Proceedings of a Workshop-in Brief.

[36] Corella D., Ordovas J.M. (2014). Aging and cardiovascular diseases: The role of gene-diet interactions. Ageing Res. Rev..

[37] Hua J., Malinski T. (2019). Variable Effects Of LDL Subclasses Of Cholesterol On Endothelial Nitric Oxide/Peroxynitrite Balance—The Risks And Clinical Implications For Cardiovascular Disease. Int. J. Nanomed..

[38] Krauss R.M. (2022). Small dense low-density lipoprotein particles: Clinically relevant?. Curr. Opin. Lipidol..

[39] Ahmed H.M., Miller M., Nasir K., McEvoy J.W., Herrington D., Blumenthal R.S., Blaha M.J. (2016). Primary Low Level of High-Density Lipoprotein Cholesterol and Risks of Coronary Heart Disease, Cardiovascular Disease, and Death: Results From the Multi-Ethnic Study of Atherosclerosis. Am. J. Epidemiol..

[40] Rosenson R.S., Brewer H.B., Ansell B.J., Barter P., Chapman M.J., Heinecke J.W., Kontush A., Tall A.R., Webb N.R. (2016). Dysfunctional HDL and atherosclerotic cardiovascular disease. Nat. Rev. Cardiol..

[41] Huang Y.Q., Liu X.C., Lo K., Liu L., Yu Y.L., Chen C.L., Huang J.Y., Feng Y.Q., Zhang B. (2020). The U Shaped Relationship Between High-Density Lipoprotein Cholesterol and All-Cause or Cause-Specific Mortality in Adult Population. Clin. Interv. Aging.

[42] Cho K.H. (2022). The Current Status of Research on High-Density Lipoproteins (HDL): A Paradigm Shift from HDL Quantity to HDL Quality and HDL Functionality. Int. J. Mol. Sci..

[43] Fernandez M.L., Webb D. (2008). The LDL to HDL cholesterol ratio as a valuable tool to evaluate coronary heart disease risk. J. Am. Coll. Nutr..

[44] Walldius G., Jungner I. (2006). The apoB/apoA-I ratio: A strong, new risk factor for cardiovascular disease and a target for lipid-lowering therapy—A review of the evidence. J. Intern. Med..

[45] Faergeman O. (2006). Introduction: Apolipoproteins and guidelines for prevention of cardiovascular disease. J. Intern. Med..

[46] Talayero B.G., Sacks F.M. (2011). The role of triglycerides in atherosclerosis. Curr. Cardiol. Rep..

[47] Murguia-Romero M., Jimenez-Flores J.R., Sigrist-Flores S.C., Espinoza-Camacho M.A., Jimenez-Morales M., Pina E., Mendez-Cruz A.R., Villalobos-Molina R., Reaven G.M. (2013). Plasma triglyceride/HDL-cholesterol ratio, insulin resistance, and cardiometabolic risk in young adults. J. Lipid Res..

[48] Quesada J.A., Bertomeu-Gonzalez V., Orozco-Beltran D., Cordero A., Gil-Guillen V.F., Lopez-Pineda A., Nouni-Garcia R., Carratala-Munuera C. (2023). The benefits of measuring the size and number of lipoprotein particles for cardiovascular risk prediction: A systematic review and meta-analysis. Clin. Investig. Arterioscler..

[49] Williams P.T., Zhao X.Q., Marcovina S.M., Otvos J.D., Brown B.G., Krauss R.M. (2014). Comparison of four methods of analysis of lipoprotein particle subfractions for their association with angiographic progression of coronary artery disease. Atherosclerosis.

[50] Emeasoba E.U., Ibeson E., Nwosu I., Montemarano N., Shani J., Shetty V.S. (2022). Clinical Relevance of Nuclear Magnetic Resonance LipoProfile. Front. Nucl. Med..

[51] Shiffman D., Louie J.Z., Caulfield M.P., Nilsson P.M., Devlin J.J., Melander O. (2017). LDL subfractions are associated with incident cardiovascular disease in the Malmo Prevention Project Study. Atherosclerosis.

[52] Lee H.C., Akhmedov A., Chen C.H. (2022). Spotlight on very-low-density lipoprotein as a driver of cardiometabolic disorders: Implications for disease progression and mechanistic insights. Front. Cardiovasc. Med..

[53] Zeb I., Jorgensen N.W., Blumenthal R.S., Burke G.L., Lloyd-Jones D., Blaha M.J., Wong N.D., Nasir K., Budoff M.J. (2021). Association of inflammatory markers and lipoprotein particle subclasses with progression of coronary artery calcium: The multi-ethnic study of atherosclerosis. Atherosclerosis.

[54] Mackey R.H., Kuller L.H., Sutton-Tyrrell K., Evans R.W., Holubkov R., Matthews K.A. (2002). Lipoprotein subclasses and coronary artery calcium in postmenopausal women from the healthy women study. Am. J. Cardiol..

[55] Shen M.Y., Hsu J.F., Chen F.Y., Lu J., Chang C.M., Madjid M., Dean J., Dixon R.A.F., Shayani S., Chou T.C. (2019). Combined LDL and VLDL Electronegativity Correlates with Coronary Heart Disease Risk in Asymptomatic Individuals. J. Clin. Med..

[56] Mora S., Otvos J.D., Rosenson R.S., Pradhan A., Buring J.E., Ridker P.M. (2010). Lipoprotein particle size and concentration by nuclear magnetic resonance and incident type 2 diabetes in women. Diabetes.

[57] Kontush A. (2015). HDL particle number and size as predictors of cardiovascular disease. Front. Pharmacol..

[58] Lund-Katz S., Phillips M.C. (2010). High density lipoprotein structure-function and role in reverse cholesterol transport. Cholesterol Binding and Cholesterol Transport Proteins.

[59] Jomard A., Osto E. (2020). High Density Lipoproteins: Metabolism, Function, and Therapeutic Potential. Front. Cardiovasc. Med..

[60] Davidson W.S., Shah A.S., Sexmith H., Gordon S.M. (2022). The HDL Proteome Watch: Compilation of studies leads to new insights on HDL function. Biochim. Biophys. Acta. Mol. Cell Biol. Lipids.

[61] Rehberger Likozar A., Zavrtanik M., Sebestjen M. (2020). Lipoprotein(a) in atherosclerosis: From pathophysiology to clinical relevance and treatment options. Ann. Med..

[62] Yanai H., Adachi H., Hakoshima M., Katsuyama H. (2022). Atherogenic Lipoproteins for the Statin Residual Cardiovascular Disease Risk. Int. J. Mol. Sci..

[63] van der Valk F.M., Bekkering S., Kroon J., Yeang C., Van den Bossche J., van Buul J.D., Ravandi A., Nederveen A.J., Verberne H.J., Scipione C. (2016). Oxidized Phospholipids on Lipoprotein(a) Elicit Arterial Wall Inflammation and an Inflammatory Monocyte Response in Humans. Circulation.

[64] Boerwinkle E., Leffert C.C., Lin J., Lackner C., Chiesa G., Hobbs H.H. (1992). Apolipoprotein(a) gene accounts for greater than 90% of the variation in plasma lipoprotein(a) concentrations. J. Clin. Investig..

[65] Mensink R.P., Katan M.B. (1990). Effect of dietary trans fatty acids on high-density and low-density lipoprotein cholesterol levels in healthy subjects. N. Engl. J. Med..

[66] Parthasarathy S., Raghavamenon A., Garelnabi M.O., Santanam N. (2010). Oxidized low-density lipoprotein. Methods Mol. Biol..

[67] Dhingra R., Vasan R.S. (2017). Biomarkers in cardiovascular disease: Statistical assessment and section on key novel heart failure biomarkers. Trends Cardiovasc. Med..

[68] Jung E., Kong S.Y., Ro Y.S., Ryu H.H., Shin S.D. (2022). Serum Cholesterol Levels and Risk of Cardiovascular Death: A Systematic Review and a Dose-Response Meta-Analysis of Prospective Cohort Studies. Int. J. Environ. Res. Public Health.

[69] Jeong S.M., Choi S., Kim K., Kim S.M., Lee G., Park S.Y., Kim Y.Y., Son J.S., Yun J.M., Park S.M. (2018). Effect of Change in Total Cholesterol Levels on Cardiovascular Disease Among Young Adults. J. Am. Heart Assoc..

[70] Mortensen M.B., Dzaye O., Botker H.E., Jensen J.M., Maeng M., Bentzon J.F., Kanstrup H., Sorensen H.T., Leipsic J., Blankstein R. (2023). Low-Density Lipoprotein Cholesterol Is Predominantly Associated With Atherosclerotic Cardiovascular Disease Events in Patients With Evidence of Coronary Atherosclerosis: The Western Denmark Heart Registry. Circulation.

[71] Liu C., Dhindsa D., Almuwaqqat Z., Ko Y.A., Mehta A., Alkhoder A.A., Alras Z., Desai S.R., Patel K.J., Hooda A. (2022). Association Between High-Density Lipoprotein Cholesterol Levels and Adverse Cardiovascular Outcomes in High-risk Populations. JAMA Cardiol..

[72] Hutchins P.M., Heinecke J.W. (2015). Cholesterol efflux capacity, macrophage reverse cholesterol transport and cardioprotective HDL. Curr. Opin. Lipidol..

[73] Khera A.V., Cuchel M., de la Llera-Moya M., Rodrigues A., Burke M.F., Jafri K., French B.C., Phillips J.A., Mucksavage M.L., Wilensky R.L. (2011). Cholesterol efflux capacity, high-density lipoprotein function, and atherosclerosis. N. Engl. J. Med..

[74] Sun T., Chen M., Shen H., PingYin, Fan L., Chen X., Wu J., Xu Z., Zhang J. (2022). Predictive value of LDL/HDL ratio in coronary atherosclerotic heart disease. BMC Cardiovasc. Disord..

[75] Samuel C., Park J., Sajja A., Michos E.D., Blumenthal R.S., Jones S.R., Martin S.S. (2023). Accuracy of 23 Equations for Estimating LDL Cholesterol in a Clinical Laboratory Database of 5,051,467 Patients. Glob. Heart.

[76] Yu Y., Li M., Huang X., Zhou W., Wang T., Zhu L., Ding C., Tao Y., Bao H., Cheng X. (2020). A U-shaped association between the LDL-cholesterol to HDL-cholesterol ratio and all-cause mortality in elderly hypertensive patients: A prospective cohort study. Lipids Health Dis..

[77] Parks E.J. (2001). Effect of dietary carbohydrate on triglyceride metabolism in humans. J. Nutr..

[78] Bookstein L., Gidding S.S., Donovan M., Smith F.A. (1990). Day-to-day variability of serum cholesterol, triglyceride, and high-density lipoprotein cholesterol levels. Impact on the assessment of risk according to the National Cholesterol Education Program guidelines. Arch. Intern. Med..

[79] Florvall G., Basu S., Larsson A. (2006). Apolipoprotein A1 is a stronger prognostic marker than are HDL and LDL cholesterol for cardiovascular disease and mortality in elderly men. J. Gerontol. Ser. A Biol. Sci. Med. Sci..

[80] Hafiane A., Genest J. (2015). High density lipoproteins: Measurement techniques and potential biomarkers of cardiovascular risk. BBA Clin..

[81] Sniderman A.D., Thanassoulis G., Glavinovic T., Navar A.M., Pencina M., Catapano A., Ference B.A. (2019). Apolipoprotein B Particles and Cardiovascular Disease: A Narrative Review. JAMA Cardiol..

[82] Behbodikhah J., Ahmed S., Elyasi A., Kasselman L.J., De Leon J., Glass A.D., Reiss A.B. (2021). Apolipoprotein B and Cardiovascular Disease: Biomarker and Potential Therapeutic Target. Metabolites.

[83] Mudd J.O., Borlaug B.A., Johnston P.V., Kral B.G., Rouf R., Blumenthal R.S., Kwiterovich P.O. (2007). Beyond low-density lipoprotein cholesterol: Defining the role of low-density lipoprotein heterogeneity in coronary artery disease. J. Am. Coll. Cardiol..

[84] Kaneva A.M., Potolitsyna N.N., Bojko E.R., Odland J.O. (2015). The apolipoprotein B/apolipoprotein A-I ratio as a potential marker of plasma atherogenicity. Dis. Markers.

[85] Sniderman A.D., Jungner I., Holme I., Aastveit A., Walldius G. (2006). Errors that result from using the TC/HDL C ratio rather than the apoB/apoA-I ratio to identify the lipoprotein-related risk of vascular disease. J. Intern. Med..

[86] Jung H.W., Hong S.P., Kim K.S. (2021). Comparison of apolipoprotein B/A1 ratio, TC/HDL-C, and lipoprotein (a) for predicting outcomes after PCI. PLoS ONE.

[87] Lawler P.R., Akinkuolie A.O., Chu A.Y., Shah S.H., Kraus W.E., Craig D., Padmanabhan L., Glynn R.J., Ridker P.M., Chasman D.I. (2017). Atherogenic Lipoprotein Determinants of Cardiovascular Disease and Residual Risk Among Individuals With Low Low-Density Lipoprotein Cholesterol. J. Am. Heart Assoc..

[88] Mora S., Szklo M., Otvos J.D., Greenland P., Psaty B.M., Goff D.C., O’Leary D.H., Saad M.F., Tsai M.Y., Sharrett A.R. (2007). LDL particle subclasses, LDL particle size, and carotid atherosclerosis in the Multi-Ethnic Study of Atherosclerosis (MESA). Atherosclerosis.

[89] Law H.G., Meyers F.J., Berglund L., Enkhmaa B. (2023). Lipoprotein(a) and diet-a challenge for a role of saturated fat in cardiovascular disease risk reduction?. Am. J. Clin. Nutr..

[90] Najjar R.S., Moore C.E., Montgomery B.D. (2018). Consumption of a defined, plant-based diet reduces lipoprotein(a), inflammation, and other atherogenic lipoproteins and particles within 4 weeks. Clin. Cardiol..

[91] Brinkley T.E., Nicklas B.J., Kanaya A.M., Satterfield S., Lakatta E.G., Simonsick E.M., Sutton-Tyrrell K., Kritchevsky S.B. (2009). Plasma oxidized low-density lipoprotein levels and arterial stiffness in older adults: The health, aging, and body composition study. Hypertension.

[92] Holvoet P., Mertens A., Verhamme P., Bogaerts K., Beyens G., Verhaeghe R., Collen D., Muls E., Van de Werf F. (2001). Circulating oxidized LDL is a useful marker for identifying patients with coronary artery disease. Arterioscler. Thromb. Vasc. Biol..

[93] Ansell B.J., Navab M., Hama S., Kamranpour N., Fonarow G., Hough G., Rahmani S., Mottahedeh R., Dave R., Reddy S.T. (2003). Inflammatory/antiinflammatory properties of high-density lipoprotein distinguish patients from control subjects better than high-density lipoprotein cholesterol levels and are favorably affected by simvastatin treatment. Circulation.

[94] Bhargava S., de la Puente-Secades S., Schurgers L., Jankowski J. (2022). Lipids and lipoproteins in cardiovascular diseases: A classification. Trends Endocrinol. Metab..

[95] van der Meer A.D., Vermeul K., Poot A.A., Feijen J., Vermes I. (2010). Flow cytometric analysis of the uptake of low-density lipoprotein by endothelial cells in microfluidic channels. Cytom. A.

[96] Rueda C.M., Rodriguez-Perea A.L., Moreno-Fernandez M., Jackson C.M., Melchior J.T., Davidson W.S., Chougnet C.A. (2017). High density lipoproteins selectively promote the survival of human regulatory T cells. J. Lipid Res..

[97] Andersen C.J., Blesso C.N., Lee J., Barona J., Shah D., Thomas M.J., Fernandez M.L. (2013). Egg Consumption Modulates HDL Lipid Composition and Increases the Cholesterol-Accepting Capacity of Serum in Metabolic Syndrome. Lipids.

[98] Zwaka T.P., Hombach V., Torzewski J. (2001). C-reactive protein-mediated low density lipoprotein uptake by macrophages: Implications for atherosclerosis. Circulation.

[99] Chemello K., Beeske S., Trang Tran T.T., Blanchard V., Villard E.F., Poirier B., Le Bail J.C., Dargazanli G., Ho-Van-Guimbal S., Boulay D. (2020). Lipoprotein(a) Cellular Uptake Ex Vivo and Hepatic Capture In Vivo Is Insensitive to PCSK9 Inhibition With Alirocumab. JACC Basic. Transl. Sci..

[100] Schoch L., Badimon L., Vilahur G. (2021). Unraveling the Complexity of HDL Remodeling: On the Hunt to Restore HDL Quality. Biomedicines.

[101] Andersen C.J. (2018). Impact of Dietary Cholesterol on the Pathophysiology of Infectious and Autoimmune Disease. Nutrients.

[102] Ronsein G.E., Vaisar T. (2017). Inflammation, remodeling, and other factors affecting HDL cholesterol efflux. Curr. Opin. Lipidol..

[103] Orekhov A.N., Nikiforov N.G., Sukhorukov V.N., Kubekina M.V., Sobenin I.A., Wu W.K., Foxx K.K., Pintus S., Stegmaier P., Stelmashenko D. (2020). Role of Phagocytosis in the Pro-Inflammatory Response in LDL-Induced Foam Cell Formation; a Transcriptome Analysis. Int. J. Mol. Sci..

[104] Van Lenten B.J., Wagner A.C., Navab M., Anantharamaiah G.M., Hama S., Reddy S.T., Fogelman A.M. (2007). Lipoprotein inflammatory properties and serum amyloid A levels but not cholesterol levels predict lesion area in cholesterol-fed rabbits. J. Lipid Res..

[105] Zhang H., Liang B., Li T., Zhou Y., Shang D., Du Z. (2018). Orexin A Suppresses Oxidized LDL Induced Endothelial Cell Inflammation via MAPK p38 and NF-kappaB Signaling Pathway. IUBMB Life.

[106] Andersen C.J., Dupree L., Murray K., Ragonesi N., McMullen K., Cintron-Rivera L., Doerr A. (2020). Low-Density Lipoproteins, High-Density Lipoproteins (HDL), and HDL-Associated Proteins Differentially Modulate Chronic Myelogenous Leukemia Cell Viability. Lipids.

[107] Navab M., Hama S.Y., Hough G.P., Subbanagounder G., Reddy S.T., Fogelman A.M. (2001). A cell-free assay for detecting HDL that is dysfunctional in preventing the formation of or inactivating oxidized phospholipids. J. Lipid Res..

[108] Guirgis F.W., Dodani S., Leeuwenburgh C., Moldawer L., Bowman J., Kalynych C., Grijalva V., Reddy S.T., Jones A.E., Moore F.A. (2018). HDL inflammatory index correlates with and predicts severity of organ failure in patients with sepsis and septic shock. PLoS ONE.

[109] van Tits L.J., Stienstra R., van Lent P.L., Netea M.G., Joosten L.A., Stalenhoef A.F. (2011). Oxidized LDL enhances pro-inflammatory responses of alternatively activated M2 macrophages: A crucial role for Kruppel-like factor 2. Atherosclerosis.

[110] Vaziri N.D., Moradi H., Pahl M.V., Fogelman A.M., Navab M. (2009). In vitro stimulation of HDL anti-inflammatory activity and inhibition of LDL pro-inflammatory activity in the plasma of patients with end-stage renal disease by an apoA-1 mimetic peptide. Kidney Int..

[111] Scoccia A.E., Molinuevo M.S., McCarthy A.D., Cortizo A.M. (2001). A simple method to assess the oxidative susceptibility of low density lipoproteins. BMC Clin. Pathol..

[112] Hargrove R.L., Etherton T.D., Pearson T.A., Harrison E.H., Kris-Etherton P.M. (2001). Low fat and high monounsaturated fat diets decrease human low density lipoprotein oxidative susceptibility in vitro. J. Nutr..

[113] de Juan-Franco E., Perez A., Ribas V., Sanchez-Hernandez J.A., Blanco-Vaca F., Ordonez-Llanos J., Sanchez-Quesada J.L. (2009). Standardization of a method to evaluate the antioxidant capacity of high-density lipoproteins. Int. J. Biomed. Sci..

[114] Karami S., Poustchi H., Sarmadi N., Radmard A.R., Ali Yari F., Pakdel A., Shabani P. (2021). Association of anti-oxidative capacity of HDL with subclinical atherosclerosis in subjects with and without non-alcoholic fatty liver disease. Diabetol. Metab. Syndr..

[115] Mahdy Ali K., Wonnerth A., Huber K., Wojta J. (2012). Cardiovascular disease risk reduction by raising HDL cholesterol--current therapies and future opportunities. Br. J. Pharmacol..

[116] Campos A.L., Sawada M., Santana M.F.M., Iborra R.T., de Assis S.I.S., Reis M., de Carvalho J.X., Gebrim L.H., Passarelli M. (2023). The increased antioxidant action of HDL is independent of HDL cholesterol plasma levels in triple-negative breast cancer. Front. Oncol..

[117] Chowaniec Z., Skoczynska A. (2018). Plasma lipid transfer proteins: The role of PLTP and CETP in atherogenesis. Adv. Clin. Exp. Med..

[118] Hovingh G.K., Hutten B.A., Holleboom A.G., Petersen W., Rol P., Stalenhoef A., Zwinderman A.H., de Groot E., Kastelein J.J., Kuivenhoven J.A. (2005). Compromised LCAT function is associated with increased atherosclerosis. Circulation.

[119] Yang K., Wang J., Xiang H., Ding P., Wu T., Ji G. (2022). LCAT-targeted therapies: Progress, failures and future. Biomed. Pharmacother..

[120] Kawano K., Qin S.C., Lin M., Tall A.R., Jiang X.C. (2000). Cholesteryl ester transfer protein and phospholipid transfer protein have nonoverlapping functions in vivo. J. Biol. Chem..

[121] Fistrek Prlic M., Coric M., Calabresi L., Pavanello C., Mosca L., Cavallari U., Vukovic Brinar I., Karanovic S., Laganovic M., Jelakovic B. (2022). Two novel variants in the lecithin:cholesterol acyltransferase gene resulted in classic LCAT deficiency. Atheroscler. Plus.

[122] Nordestgaard L.T., Christoffersen M., Lauridsen B.K., Afzal S., Nordestgaard B.G., Frikke-Schmidt R., Tybjaerg-Hansen A. (2022). Long-term Benefits and Harms Associated With Genetic Cholesteryl Ester Transfer Protein Deficiency in the General Population. JAMA Cardiol..

[123] Vergeer M., Boekholdt S.M., Sandhu M.S., Ricketts S.L., Wareham N.J., Brown M.J., de Faire U., Leander K., Gigante B., Kavousi M. (2010). Genetic variation at the phospholipid transfer protein locus affects its activity and high-density lipoprotein size and is a novel marker of cardiovascular disease susceptibility. Circulation.

[124] Schlitt A., Blankenberg S., Bickel C., Lackner K.J., Heine G.H., Buerke M., Werdan K., Maegdefessel L., Raaz U., Rupprecht H.J. (2009). PLTP activity is a risk factor for subsequent cardiovascular events in CAD patients under statin therapy: The AtheroGene study. J. Lipid Res..

[125] Vasan R.S., Pencina M.J., Robins S.J., Zachariah J.P., Kaur G., D’Agostino R.B., Ordovas J.M. (2009). Association of circulating cholesteryl ester transfer protein activity with incidence of cardiovascular disease in the community. Circulation.

[126] Solajic-Bozicevic N., Stavljenic-Rukavina A., Sesto M. (1994). Lecithin-cholesterol acryltransferase activity in patients with coronary artery disease examined by coronary angiography. Clin. Investig..

[127] Solajic-Bozicevic N., Stavljenic A., Sesto M. (1991). Lecithin:cholesterol acyltransferase activity in patients with acute myocardial infarction and coronary heart disease. Artery.

[128] Rousset X., Shamburek R., Vaisman B., Amar M., Remaley A.T. (2011). Lecithin cholesterol acyltransferase: An anti- or pro-atherogenic factor?. Curr. Atheroscler. Rep..

[129] Feng J., Wang Y., Li W., Zhao Y., Liu Y., Yao X., Liu S., Yu P., Li R. (2022). High levels of oxidized fatty acids in HDL impair the antioxidant function of HDL in patients with diabetes. Front. Endocrinol..

[130] Nicholls S.J., Ditmarsch M., Kastelein J.J., Rigby S.P., Kling D., Curcio D.L., Alp N.J., Davidson M.H. (2022). Lipid lowering effects of the CETP inhibitor obicetrapib in combination with high-intensity statins: A randomized phase 2 trial. Nat. Med..

[131] Schmidt A.F., Hunt N.B., Gordillo-Maranon M., Charoen P., Drenos F., Kivimaki M., Lawlor D.A., Giambartolomei C., Papacosta O., Chaturvedi N. (2021). Cholesteryl ester transfer protein (CETP) as a drug target for cardiovascular disease. Nat. Commun..

[132] Schgoer W., Mueller T., Jauhiainen M., Wehinger A., Gander R., Tancevski I., Salzmann K., Eller P., Ritsch A., Haltmayer M. (2008). Low phospholipid transfer protein (PLTP) is a risk factor for peripheral atherosclerosis. Atherosclerosis.

[133] Babu M., Snyder M. (2023). Multi-Omics Profiling for Health. Mol. Cell Proteom..

[134] Palstrom N.B., Matthiesen R., Rasmussen L.M., Beck H.C. (2022). Recent Developments in Clinical Plasma Proteomics-Applied to Cardiovascular Research. Biomedicines.

[135] Schissel S.L., Tweedie-Hardman J., Rapp J.H., Graham G., Williams K.J., Tabas I. (1996). Rabbit aorta and human atherosclerotic lesions hydrolyze the sphingomyelin of retained low-density lipoprotein. Proposed role for arterial-wall sphingomyelinase in subendothelial retention and aggregation of atherogenic lipoproteins. J. Clin. Investig..

[136] Kostara C.E., Papathanasiou A., Cung M.T., Elisaf M.S., Goudevenos J., Bairaktari E.T. (2010). Evaluation of established coronary heart disease on the basis of HDL and non-HDL NMR lipid profiling. J. Proteome Res..

[137] Kostara C.E., Papathanasiou A., Psychogios N., Cung M.T., Elisaf M.S., Goudevenos J., Bairaktari E.T. (2014). NMR-based lipidomic analysis of blood lipoproteins differentiates the progression of coronary heart disease. J. Proteome Res..

[138] Sutter I., Velagapudi S., Othman A., Riwanto M., Manz J., Rohrer L., Rentsch K., Hornemann T., Landmesser U., von Eckardstein A. (2015). Plasmalogens of high-density lipoproteins (HDL) are associated with coronary artery disease and anti-apoptotic activity of HDL. Atherosclerosis.

[139] Meikle P.J., Formosa M.F., Mellett N.A., Jayawardana K.S., Giles C., Bertovic D.A., Jennings G.L., Childs W., Reddy M., Carey A.L. (2019). HDL Phospholipids, but Not Cholesterol Distinguish Acute Coronary Syndrome From Stable Coronary Artery Disease. J. Am. Heart Assoc..

[140] Cardner M., Yalcinkaya M., Goetze S., Luca E., Balaz M., Hunjadi M., Hartung J., Shemet A., Krankel N., Radosavljevic S. (2020). Structure-function relationships of HDL in diabetes and coronary heart disease. JCI Insight.

[141] Elhadad M.A., Del C.G.-A.M., Chen C.W., Neumeyer S., Delerue T., Rathmann W., Nabauer M., Meisinger C., Kaab S., Seissler J. (2024). Plasma proteome association with coronary heart disease and carotid intima media thickness: Results from the KORA F4 study. Cardiovasc. Diabetol..

[142] von Zychlinski A., Kleffman T. (2015). Dissecting the proteome of lipoproteins: New biomarkers for cardiovascular diseases?. Transl. Proteom..

[143] Bourgeois R., Girard A., Perrot N., Guertin J., Mitchell P.L., Couture C., Gotti C., Bourassa S., Poggio P., Mass E. (2021). A Comparative Analysis of the Lipoprotein(a) and Low-Density Lipoprotein Proteomic Profiles Combining Mass Spectrometry and Mendelian Randomization. CJC Open.

[144] Karlsson H., Mortstedt H., Lindqvist H., Tagesson C., Lindahl M. (2009). Protein profiling of low-density lipoprotein from obese subjects. Proteom. Clin. Appl..

[145] Lepedda A.J., Nieddu G., Zinellu E., De Muro P., Piredda F., Guarino A., Spirito R., Carta F., Turrini F., Formato M. (2013). Proteomic analysis of plasma-purified VLDL, LDL, and HDL fractions from atherosclerotic patients undergoing carotid endarterectomy: Identification of serum amyloid A as a potential marker. Oxid. Med. Cell Longev..

[146] Alwaili K., Bailey D., Awan Z., Bailey S.D., Ruel I., Hafiane A., Krimbou L., Laboissiere S., Genest J. (2012). The HDL proteome in acute coronary syndromes shifts to an inflammatory profile. Biochim. Biophys. Acta.

[147] Plubell D.L., Fenton A.M., Rosario S., Bergstrom P., Wilmarth P.A., Clark W.M., Zakai N.A., Quinn J.F., Minnier J., Alkayed N.J. (2020). High-Density Lipoprotein Carries Markers That Track With Recovery From Stroke. Circ. Res..

[148] Mao J.Y., Sun J.T., Yang K., Shen W.F., Lu L., Zhang R.Y., Tong X., Liu Y. (2017). Serum amyloid A enrichment impairs the anti-inflammatory ability of HDL from diabetic nephropathy patients. J. Diabetes Complicat..

[149] Gourgari E., Ma J., Playford M.P., Mehta N.N., Goldman R., Remaley A.T., Gordon S.M. (2019). Proteomic alterations of HDL in youth with type 1 diabetes and their associations with glycemic control: A case-control study. Cardiovasc. Diabetol..

[150] Watanabe J., Charles-Schoeman C., Miao Y., Elashoff D., Lee Y.Y., Katselis G., Lee T.D., Reddy S.T. (2012). Proteomic profiling following immunoaffinity capture of high-density lipoprotein: Association of acute-phase proteins and complement factors with proinflammatory high-density lipoprotein in rheumatoid arthritis. Arthritis Rheum..

[151] Holzer M., Wolf P., Curcic S., Birner-Gruenberger R., Weger W., Inzinger M., El-Gamal D., Wadsack C., Heinemann A., Marsche G. (2012). Psoriasis alters HDL composition and cholesterol efflux capacity. J. Lipid Res..

[152] Chapman M.J., Orsoni A., Mellett N.A., Nguyen A., Robillard P., Shaw J.E., Giral P., Therond P., Swertfeger D., Davidson W.S. (2024). Pitavastatin treatment remodels the HDL subclass lipidome and proteome in hypertriglyceridemia. J. Lipid Res..

[153] Yang K., Han X. (2016). Lipidomics: Techniques, Applications, and Outcomes Related to Biomedical Sciences. Trends Biochem. Sci..

[154] Christinat N., Masoodi M. (2017). Comprehensive Lipoprotein Characterization Using Lipidomics Analysis of Human Plasma. J. Proteome Res..

[155] Kontush A., Lhomme M., Chapman M.J. (2013). Unraveling the complexities of the HDL lipidome. J. Lipid Res..

[156] Andersen C.J., Huang L., Zhai F., Esposito C.P., Greco J.M., Zhang R., Woodruff R., Sloan A., Van Dyke A.R. (2023). Consumption of Different Egg-Based Diets Alters Clinical Metabolic and Hematological Parameters in Young, Healthy Men and Women. Nutrients.

[157] Sawrey-Kubicek L., Zhu C., Bardagjy A.S., Rhodes C.H., Sacchi R., Randolph J.M., Steinberg F.M., Zivkovic A.M. (2019). Whole egg consumption compared with yolk-free egg increases the cholesterol efflux capacity of high-density lipoproteins in overweight, postmenopausal women. Am. J. Clin. Nutr..

[158] Fernandez-Castillejo S., Pedret A., Catalan U., Valls R.M., Farras M., Rubio L., Castaner O., Macia A., Fito M., Motilva M.J. (2021). Virgin Olive Oil Phenolic Compounds Modulate the HDL Lipidome in Hypercholesterolaemic Subjects: A Lipidomic Analysis of the VOHF Study. Mol. Nutr. Food Res..

[159] Zhu C., Sawrey-Kubicek L., Beals E., Hughes R.L., Rhodes C.H., Sacchi R., Zivkovic A.M. (2019). The HDL lipidome is widely remodeled by fast food versus Mediterranean diet in 4 days. Metabolomics.

[160] McGregor E., Dunn M.J. (2003). Proteomics of heart disease. Hum. Mol. Genet..

[161] Chen H., Miao H., Feng Y.L., Zhao Y.Y., Lin R.C. (2014). Metabolomics in dyslipidemia. Adv. Clin. Chem..

[162] Christians U., Schmitz V., Klawitter J., Klawitter J. (2012). Proteo-Metabolomic Strategies in the Future of Drug Development. Analytical Techniques for Clinical Chemistry.

[163] Aebersold R., Mann M. (2003). Mass spectrometry-based proteomics. Nature.

[164] Li R.X., Ding Y.B., Zhao S.L., Xiao Y.Y., Li Q.R., Xia F.Y., Sun L., Lin X., Wu J.R., Liao K. (2012). Secretome-derived isotope tags (SDIT) reveal adipocyte-derived apolipoprotein C-I as a predictive marker for cardiovascular disease. J. Proteome Res..

[165] Kaga E., Karademir B., Baykal A.T., Ozer N.K. (2013). Identification of differentially expressed proteins in atherosclerotic aorta and effect of vitamin E. J. Proteom..

[166] Tenger C., Zhou X. (2003). Apolipoprotein E modulates immune activation by acting on the antigen-presenting cell. Immunology.

[167] Wilhelm A.J., Zabalawi M., Owen J.S., Shah D., Grayson J.M., Major A.S., Bhat S., Gibbs D.P., Thomas M.J., Sorci-Thomas M.G. (2010). Apolipoprotein A-I modulates regulatory T cells in autoimmune LDLr^−/−^, ApoA-I^−/−^ mice. J. Biol. Chem..

[168] Lv Y., Tang W., Xu Y., Chang W., Zhang Z., Lin Q., Ji M., Feng Q., He G., Xu J. (2023). Apolipoprotein L3 enhances CD8+ T cell antitumor immunity of colorectal cancer by promoting LDHA-mediated ferroptosis. Int. J. Biol. Sci..

[169] van den Elzen P., Garg S., Leon L., Brigl M., Leadbetter E.A., Gumperz J.E., Dascher C.C., Cheng T.Y., Sacks F.M., Illarionov P.A. (2005). Apolipoprotein-mediated pathways of lipid antigen presentation. Nature.

[170] Getz G.S., Reardon C.A. (2019). Apoproteins E, A-I, and SAA in Macrophage Pathobiology Related to Atherogenesis. Front. Pharmacol..

[171] Gordon S.M., Chung J.H., Playford M.P., Dey A.K., Sviridov D., Seifuddin F., Chen Y.C., Pirooznia M., Chen M.Y., Mehta N.N. (2018). High density lipoprotein proteome is associated with cardiovascular risk factors and atherosclerosis burden as evaluated by coronary CT angiography. Atherosclerosis.

[172] Davidson W.S. HDL Proteome Watch. https://homepages.uc.edu/~davidswm/HDLproteome.html.

[173] Davidson W.S. LDL Proteome Watch. https://homepages.uc.edu/~davidswm/LDLproteome.html.

[174] Head R.J., Buckley J.D. (2020). Human variation in response to food and nutrients. Nutr. Rev..

[175] Xu L.B., Zhou Y.F., Yao J.L., Sun S.J., Rui Q., Yang X.J., Li X.B. (2017). Apolipoprotein A1 polymorphisms and risk of coronary artery disease: A meta-analysis. Arch. Med. Sci..

[176] Ordovas J.M. (2009). Genetic influences on blood lipids and cardiovascular disease risk: Tools for primary prevention. Am. J. Clin. Nutr..

[177] Wang J., Xiao Q., Wang L., Wang Y., Wang D., Ding H. (2022). Role of ABCA1 in Cardiovascular Disease. J. Pers. Med..

[178] Oldoni F., Baldassarre D., Castelnuovo S., Ossoli A., Amato M., van Capelleveen J., Hovingh G.K., De Groot E., Bochem A., Simonelli S. (2018). Complete and Partial Lecithin:Cholesterol Acyltransferase Deficiency Is Differentially Associated with Atherosclerosis. Circulation.

[179] Ha E.E., Van Camp A.G., Bauer R.C. (2019). Genetics-driven discovery of novel regulators of lipid metabolism. Curr. Opin. Lipidol..

[180] Tall A.R., Rader D.J. (2018). Trials and Tribulations of CETP Inhibitors. Circ. Res..

[181] Arca M., Celant S., Olimpieri P.P., Colatrella A., Tomassini L., D’Erasmo L., Averna M., Zambon A., Catapano A.L., Russo P. (2023). Real-World Effectiveness of PCSK9 Inhibitors in Reducing LDL-C in Patients With Familial Hypercholesterolemia in Italy: A Retrospective Cohort Study Based on the AIFA Monitoring Registries. J. Am. Heart Assoc..

[182] Shi Y., Zhang H., Huang S., Yin L., Wang F., Luo P., Huang H. (2022). Epigenetic regulation in cardiovascular disease: Mechanisms and advances in clinical trials. Signal Transduct. Target Ther..

[183] Alagarsamy J., Jaeschke A., Hui D.Y. (2022). Apolipoprotein E in Cardiometabolic and Neurological Health and Diseases. Int. J. Mol. Sci..

[184] Song Y., Stampfer M.J., Liu S. (2004). Meta-analysis: Apolipoprotein E genotypes and risk for coronary heart disease. Ann. Intern. Med..

[185] Mahley R.W., Weisgraber K.H., Huang Y. (2009). Apolipoprotein E: Structure determines function, from atherosclerosis to Alzheimer’s disease to AIDS. J. Lipid Res..

[186] Dong L.M., Weisgraber K.H. (1996). Human apolipoprotein E4 domain interaction. Arginine 61 and glutamic acid 255 interact to direct the preference for very low density lipoproteins. J. Biol. Chem..

[187] Deane R., Sagare A., Zlokovic B.V. (2008). The role of the cell surface LRP and soluble LRP in blood-brain barrier Abeta clearance in Alzheimer’s disease. Curr. Pharm. Des..

[188] Vezeridis A.M., Chroni A., Zannis V.I. (2011). Domains of apoE4 required for the biogenesis of apoE-containing HDL. Ann. Med..

[189] Hong B.V., Zheng J., Agus J.K., Tang X., Lebrilla C.B., Jin L.W., Maezawa I., Erickson K., Harvey D.J., DeCarli C.S. (2022). High-Density Lipoprotein Changes in Alzheimer’s Disease Are APOE Genotype-Specific. Biomedicines.

[190] de Rojas I., Del Barrio L., Hernandez I., Montrreal L., Garcia-Gonzalez P., Marquie M., Valero S., Cano A., Orellana A., Boada M. (2023). Correlations between the NMR Lipoprotein Profile, APOE Genotype, and Cholesterol Efflux Capacity of Fasting Plasma from Cognitively Healthy Elderly Adults. Int. J. Mol. Sci..

[191] Shah N.P., Ahmed H.M., Wilson Tang W.H. (2020). Familial hypercholesterolemia: Detect, treat, and ask about family. Cleve. Clin. J. Med..

[192] Collaboration E.A.S.F.H.S., Vallejo-Vaz A.J., Akram A., Kondapally Seshasai S.R., Cole D., Watts G.F., Hovingh G.K., Kastelein J.J., Mata P., Raal F.J. (2016). Pooling and expanding registries of familial hypercholesterolaemia to assess gaps in care and improve disease management and outcomes: Rationale and design of the global EAS Familial Hypercholesterolaemia Studies Collaboration. Atheroscler. Suppl..

[193] Huijgen R., Kindt I., Defesche J.C., Kastelein J.J. (2012). Cardiovascular risk in relation to functionality of sequence variants in the gene coding for the low-density lipoprotein receptor: A study among 29,365 individuals tested for 64 specific low-density lipoprotein-receptor sequence variants. Eur. Heart J..

[194] Sturm A.C., Knowles J.W., Gidding S.S., Ahmad Z.S., Ahmed C.D., Ballantyne C.M., Baum S.J., Bourbon M., Carrie A., Cuchel M. (2018). Clinical Genetic Testing for Familial Hypercholesterolemia: JACC Scientific Expert Panel. J. Am. Coll. Cardiol..

[195] Goldstein J.L., Brown M.S. (2009). The LDL receptor. Arterioscler. Thromb. Vasc. Biol..

[196] Rodriguez-Jimenez C., de la Pena G., Sanguino J., Poyatos-Pelaez S., Carazo A., Martinez-Hernandez P.L., Arrieta F., Mostaza J.M., Gomez-Coronado D., Rodriguez-Novoa S. (2023). Identification and Functional Analysis of APOB Variants in a Cohort of Hypercholesterolemic Patients. Int. J. Mol. Sci..

[197] Abifadel M., Varret M., Rabes J.P., Allard D., Ouguerram K., Devillers M., Cruaud C., Benjannet S., Wickham L., Erlich D. (2003). Mutations in PCSK9 cause autosomal dominant hypercholesterolemia. Nat. Genet..

[198] Kent S.T., Rosenson R.S., Avery C.L., Chen Y.I., Correa A., Cummings S.R., Cupples L.A., Cushman M., Evans D.S., Gudnason V. (2017). PCSK9 Loss-of-Function Variants, Low-Density Lipoprotein Cholesterol, and Risk of Coronary Heart Disease and Stroke: Data From 9 Studies of Blacks and Whites. Circ. Cardiovasc. Genet..

[199] Guo Q., Feng X., Zhou Y. (2020). PCSK9 Variants in Familial Hypercholesterolemia: A Comprehensive Synopsis. Front. Genet..

[200] Imbalzano E., Ilardi F., Orlando L., Pintaudi B., Savarese G., Rosano G. (2023). The efficacy of PCSK9 inhibitors on major cardiovascular events and lipid profile in patients with diabetes: A systematic review and meta-analysis of randomized controlled trials. Eur. Heart J. Cardiovasc. Pharmacother..

[201] Jellinger P.S., Handelsman Y., Rosenblit P.D., Bloomgarden Z.T., Fonseca V.A., Garber A.J., Grunberger G., Guerin C.K., Bell D.S.H., Mechanick J.I. (2017). American Association of Clinical Endocrinologists and American College of Endocrinology Guidelines for Management of Dyslipidemia and Prevention of Cardiovascular Disease. Endocr. Pract..

[202] Ganjali S., Hosseini S., Rizzo M., Kontush A., Sahebkar A. (2023). Capacity of HDL to Efflux Cellular Cholesterol from Lipid-Loaded Macrophages Is Reduced in Patients with Familial Hypercholesterolemia. Metabolites.

[203] Darabi M., Lhomme M., Ponnaiah M., Pucic-Bakovic M., Guillas I., Frisdal E., Bittar R., Croyal M., Matheron-Duriez L., Poupel L. (2023). Integrated omics approach for the identification of HDL structure-function relationships in PCSK9-related familial hypercholesterolemia. J. Clin. Lipidol..

[204] Kim M., Long T.I., Arakawa K., Wang R., Yu M.C., Laird P.W. (2010). DNA methylation as a biomarker for cardiovascular disease risk. PLoS ONE.

[205] Aavik E., Lumivuori H., Leppanen O., Wirth T., Hakkinen S.K., Brasen J.H., Beschorner U., Zeller T., Braspenning M., van Criekinge W. (2015). Global DNA methylation analysis of human atherosclerotic plaques reveals extensive genomic hypomethylation and reactivation at imprinted locus 14q32 involving induction of a miRNA cluster. Eur. Heart J..

[206] Yamada Y., Horibe H., Oguri M., Sakuma J., Takeuchi I., Yasukochi Y., Kato K., Sawabe M. (2018). Identification of novel hyper- or hypomethylated CpG sites and genes associated with atherosclerotic plaque using an epigenome-wide association study. Int. J. Mol. Sci..

[207] Dai Y., Chen D., Xu T. (2022). DNA Methylation Aberrant in Atherosclerosis. Front. Pharmacol..

[208] Sayols-Baixeras S., Hernaez A., Subirana I., Lluis-Ganella C., Munoz D., Fito M., Marrugat J., Elosua R. (2017). DNA Methylation and High-Density Lipoprotein Functionality-Brief Report: The REGICOR Study (Registre Gironi del Cor). Arterioscler. Thromb. Vasc. Biol..

[209] Robinson E.L. (2021). Histone modifications in cardiovascular disease initiation and progression. Epigenetics in Cardiovascular Disease.

[210] Poller W., Dimmeler S., Heymans S., Zeller T., Haas J., Karakas M., Leistner D.M., Jakob P., Nakagawa S., Blankenberg S. (2018). Non-coding RNAs in cardiovascular diseases: Diagnostic and therapeutic perspectives. Eur. Heart J..

[211] Canfran-Duque A., Lin C.S., Goedeke L., Suarez Y., Fernandez-Hernando C. (2016). Micro-RNAs and High-Density Lipoprotein Metabolism. Arterioscler. Thromb. Vasc. Biol..

[212] Fernandez-Tussy P., Ruz-Maldonado I., Fernandez-Hernando C. (2021). MicroRNAs and Circular RNAs in Lipoprotein Metabolism. Curr. Atheroscler. Rep..

[213] Li X., Qi L. (2022). Epigenetics in Precision Nutrition. J. Pers. Med..

[214] Thomas M.S., Blesso C.N., Calle M.C., Chun O.K., Puglisi M., Fernandez M.L. (2022). Dietary Influences on Gut Microbiota with a Focus on Metabolic Syndrome. Metab. Syndr. Relat. Disord..

[215] Fukuda S., Ohno H. (2014). Gut microbiome and metabolic diseases. Semin. Immunopathol..

[216] Lei L., Zhao N., Zhang L., Chen J., Liu X., Piao S. (2022). Gut microbiota is a potential goalkeeper of dyslipidemia. Front. Endocrinol..

[217] Kimura I., Ozawa K., Inoue D., Imamura T., Kimura K., Maeda T., Terasawa K., Kashihara D., Hirano K., Tani T. (2013). The gut microbiota suppresses insulin-mediated fat accumulation via the short-chain fatty acid receptor GPR43. Nat. Comm..

[218] Todesco T., Rao A.V., Bosello O., Jenkins D.J. (1991). Propionate lowers blood glucose and alters lipid metabolism in healthy subjects. Am. J. Clin. Nutr..

[219] Fu J., Bonder M.J., Cenit M.C., Tigchelaar E.F., Maatman A., Dekens J.A., Brandsma E., Marczynska J., Imhann F., Weersma R.K. (2015). The Gut Microbiome Contributes to a Substantial Proportion of the Variation in Blood Lipids. Circ. Res..

[220] Thomas M.S., Fernandez M.L. (2021). Trimethylamine N-Oxide (TMAO), Diet and Cardiovascular Disease. Curr. Atheroscler. Rep..

[221] Dong Z., Liang Z., Guo M., Hu S., Shen Z., Hai X. (2018). The Association between Plasma Levels of Trimethylamine N-Oxide and the Risk of Coronary Heart Disease in Chinese Patients with or without Type 2 Diabetes Mellitus. Dis. Markers.

[222] Ding L., Chang M., Guo Y., Zhang L., Xue C., Yanagita T., Zhang T., Wang Y. (2018). Trimethylamine-N-oxide (TMAO)-induced atherosclerosis is associated with bile acid metabolism. Lipids Health Dis..

[223] Wang Z., Klipfell E., Bennett B.J., Koeth R., Levison B.S., Dugar B., Feldstein A.E., Britt E.B., Fu X., Chung Y.M. (2011). Gut flora metabolism of phosphatidylcholine promotes cardiovascular disease. Nature.

[224] Canyelles M., Tondo M., Cedo L., Farras M., Escola-Gil J.C., Blanco-Vaca F. (2018). Trimethylamine N-Oxide: A Link among Diet, Gut Microbiota, Gene Regulation of Liver and Intestine Cholesterol Homeostasis and HDL Function. Int. J. Mol. Sci..

[225] Jie Z., Yu X., Liu Y., Sun L., Chen P., Ding Q., Gao Y., Zhang X., Yu M., Liu Y. (2021). The Baseline Gut Microbiota Directs Dieting-Induced Weight Loss Trajectories. Gastroenterology.

[226] Wang Q., Pang D., Wang H. (2023). Effect of overall lifestyle on the all-cause mortality and cardiovascular disease death in dyslipidemia patients with or without lipid-lowering therapy: A cohort study. BMC Cardiovasc. Disord..

[227] Sharifi-Rad J., Rodrigues C.F., Sharopov F., Docea A.O., Can Karaca A., Sharifi-Rad M., Kahveci Karincaoglu D., Gulseren G., Senol E., Demircan E. (2020). Diet, Lifestyle and Cardiovascular Diseases: Linking Pathophysiology to Cardioprotective Effects of Natural Bioactive Compounds. Int. J. Environ. Res. Public Health.

[228] Feinman R.D., Volek J.S. (2006). Low carbohydrate diets improve atherogenic dyslipidemia even in the absence of weight loss. Nutr. Metab..

[229] Tucker L.A. (2022). Macronutrient Intake and Insulin Resistance in 5665 Randomly Selected, Non-Diabetic U.S. Adults. Nutrients.

[230] Pan F., Wang Z., Wang H., Su C., Zhang J., Du W., Jia X., Wang L., Jiang H., Li W. (2022). Association between Free Sugars Intake and Risk of Metabolic Syndrome in Chinese Adults: Results from the China Health and Nutrition Survey, 2000–2018. Nutrients.

[231] Grundy S.M., Brewer H.B., Cleeman J.I., Smith S.C., Lenfant C., American Heart A., National Heart L., Blood I. (2004). Definition of metabolic syndrome: Report of the National Heart, Lung, and Blood Institute/American Heart Association conference on scientific issues related to definition. Circulation.

[232] Willett W.C., Stampfer M.J., Manson J.E., Colditz G.A., Speizer F.E., Rosner B.A., Sampson L.A., Hennekens C.H. (1993). Intake of trans fatty acids and risk of coronary heart disease among women. Lancet.

[233] Hu F.B., Willett W.C. (2002). Optimal diets for prevention of coronary heart disease. J. Am. Med. Assoc..

[234] Baum S.J., Kris-Etherton P.M., Willett W.C., Lichtenstein A.H., Rudel L.L., Maki K.C., Whelan J., Ramsden C.E., Block R.C. (2012). Fatty acids in cardiovascular health and disease: A comprehensive update. J. Clin. Lipidol..

[235] Astrup A., Bertram H.C., Bonjour J.P., de Groot L.C., de Oliveira Otto M.C., Feeney E.L., Garg M.L., Givens I., Kok F.J., Krauss R.M. (2019). WHO draft guidelines on dietary saturated and trans fatty acids: Time for a new approach?. BMJ.

[236] Oh K., Hu F.B., Manson J.E., Stampfer M.J., Willett W.C. (2005). Dietary fat intake and risk of coronary heart disease in women: 20 years of follow-up of the nurses’ health study. Am. J. Epidemiol..

[237] Ross R. (1993). The pathogenesis of atherosclerosis: A perspective for the 1990s. Nature.

[238] Kanter M.M., Kris-Etherton P.M., Fernandez M.L., Vickers K.C., Katz D.L. (2012). Exploring the factors that affect blood cholesterol and heart disease risk: Is dietary cholesterol as bad for you as history leads us to believe?. Adv. Nutr..

[239] Fernandez M.L. (2022). The Role of Eggs in Healthy Diets. J. Fam. Pract..

[240] Missimer A., DiMarco D.M., Andersen C.J., Murillo A.G., Vergara-Jimenez M., Fernandez M.L. (2017). Consuming Two Eggs per Day, as Compared to an Oatmeal Breakfast, Decreases Plasma Ghrelin while Maintaining the LDL/HDL Ratio. Nutrients.

[241] DiMarco D.M., Missimer A., Murillo A.G., Lemos B.S., Malysheva O.V., Caudill M.A., Blesso C.N., Fernandez M.L. (2017). Intake of up to 3 Eggs/Day Increases HDL Cholesterol and Plasma Choline While Plasma Trimethylamine-N-oxide is Unchanged in a Healthy Population. Lipids.

[242] U.S. Department of Agriculture, Agricultural Research Service (2015). USDA National Nutrient Database for Standard Reference, Release 27 (Revised).

[243] Phillips J.A. (2021). Dietary Guidelines for Americans, 2020–2025. Workplace Health Saf..

[244] Murillo A.G., Fernandez M.L. (2016). Potential of Dietary Non-Provitamin A Carotenoids in the Prevention and Treatment of Diabetic Microvascular Complications. Adv. Nutr. Int. Rev. J..

[245] Bakac E.R., Percin E., Gunes-Bayir A., Dadak A. (2023). A Narrative Review: The Effect and Importance of Carotenoids on Aging and Aging-Related Diseases. Int. J. Mol. Sci..

[246] Murillo A.G., Fernandez M.L. (2017). The Relevance of Dietary Polyphenols in Cardiovascular Protection. Curr. Pharm. Dis..

[247] Cheng Y.C., Sheen J.M., Hu W.L., Hung Y.C. (2017). Polyphenols and Oxidative Stress in Atherosclerosis-Related Ischemic Heart Disease and Stroke. Oxid. Med. Cell Longev..

[248] Djuricic I., Calder P.C. (2021). Beneficial Outcomes of Omega-6 and Omega-3 Polyunsaturated Fatty Acids on Human Health: An Update for 2021. Nutrients.

[249] Andersen C.J., Fernandez M.L. (2013). DIetary approaches to improving atheroprotective HDL functions. Food Func..

[250] Villacorta L., Azzi A., Zingg J.M. (2007). Regulatory role of vitamins E and C on extracellular matrix components of the vascular system. Mol. Asp. Med..

[251] Caliri A.W., Tommasi S., Besaratinia A. (2021). Relationships among smoking, oxidative stress, inflammation, macromolecular damage, and cancer. Mutat. Res. Rev. Mutat. Res..

[252] Suzuki K., Ito Y., Ochiai J., Aoki K., Wakai K., Tamakoshi A., Ando M., Watanabe Y., Ozasa K., Seki N. (2003). The relationship between smoking habits and serum levels of 8-OHdG, oxidized LDL antibodies, Mn-SOD and carotenoids in rural Japanese residents. J. Epidemiol..

[253] Gepner A.D., Piper M.E., Johnson H.M., Fiore M.C., Baker T.B., Stein J.H. (2011). Effects of smoking and smoking cessation on lipids and lipoproteins: Outcomes from a randomized clinical trial. Am. Heart J..

[254] Zaid M., Miura K., Okayama A., Nakagawa H., Sakata K., Saitoh S., Okuda N., Yoshita K., Choudhury S.R., Rodriguez B. (2018). Associations of High-Density Lipoprotein Particle and High-Density Lipoprotein Cholesterol With Alcohol Intake, Smoking, and Body Mass Index- The INTERLIPID Study. Circ. J..

[255] Kannel W.B., Ellison R.C. (1996). Alcohol and coronary heart disease: The evidence for a protective effect. Clin. Chim. Acta..

[256] Baraona E., Lieber C.S. (1979). Effects of ethanol on lipid metabolism. J. Lipid Res..

[257] Cho K.H., Nam H.S., Kang D.J., Park M.H., Kim J.H. (2022). Long-Term Alcohol Consumption Caused a Significant Decrease in Serum High-Density Lipoprotein (HDL)-Cholesterol and Apolipoprotein A-I with the Atherogenic Changes of HDL in Middle-Aged Korean Women. Int. J. Mol. Sci..

[258] Piepoli M.F., Hoes A.W., Agewall S., Albus C., Brotons C., Catapano A.L., Cooney M.T., Corra U., Cosyns B., Deaton C. (2016). 2016 European Guidelines on cardiovascular disease prevention in clinical practice: The Sixth Joint Task Force of the European Society of Cardiology and Other Societies on Cardiovascular Disease Prevention in Clinical Practice (constituted by representatives of 10 societies and by invited experts)Developed with the special contribution of the European Association for Cardiovascular Prevention & Rehabilitation (EACPR). Eur. Heart J..

[259] Zhu J., Zhang Y., Wu Y., Xiang Y., Tong X., Yu Y., Qiu Y., Cui S., Zhao Q., Wang N. (2022). Obesity and Dyslipidemia in Chinese Adults: A Cross-Sectional Study in Shanghai, China. Nutrients.

[260] Klop B., Elte J.W., Cabezas M.C. (2013). Dyslipidemia in obesity: Mechanisms and potential targets. Nutrients.

[261] Lofgren I.E., Herron K.L., West K.L., Zern T.L., Brownbill R.A., Ilich J.Z., Koo S.I., Fernandez M.L. (2005). Weight loss favorably modifies anthropometrics and reverses the metabolic syndrome in premenopausal women. J. Am. Coll. Nutr..

[262] Magkos F., Fraterrigo G., Yoshino J., Luecking C., Kirbach K., Kelly S.C., de las Fuentes L., He S., Okunade A.L., Patterson B.W. (2016). Effects of Moderate and Subsequent Progressive Weight Loss on Metabolic Function and Adipose Tissue Biology in Humans with Obesity. Cell Metab..

[263] Krause B.R., Hartman A.D. (1984). Adipose tissue and cholesterol metabolism. J. Lipid Res..

[264] McGillicuddy F.C., Reilly M.P., Rader D.J. (2011). Adipose modulation of high-density lipoprotein cholesterol: Implications for obesity, high-density lipoprotein metabolism, and cardiovascular disease. Circulation.

[265] Mittendorfer B., Patterson B.W., Klein S. (2003). Effect of weight loss on VLDL-triglyceride and apoB-100 kinetics in women with abdominal obesity. Am. J. Physiol. Endocrinol. Metab..

[266] Powell-Wiley T.M., Poirier P., Burke L.E., Despres J.P., Gordon-Larsen P., Lavie C.J., Lear S.A., Ndumele C.E., Neeland I.J., Sanders P. (2021). Obesity and Cardiovascular Disease: A Scientific Statement From the American Heart Association. Circulation.

[267] Warren T.Y., Barry V., Hooker S.P., Sui X., Church T.S., Blair S.N. (2010). Sedentary behaviors increase risk of cardiovascular disease mortality in men. Med. Sci. Sports Exerc..

[268] Gu D., Wang D., Zhu Q., Luo L., Zhang T. (2024). Prevalence of dyslipidemia and associated factors in sedentary occupational population from Shanghai: A cross-sectional study. Arch. Public Health.

[269] Lavie C.J., Ozemek C., Carbone S., Katzmarzyk P.T., Blair S.N. (2019). Sedentary Behavior, Exercise, and Cardiovascular Health. Circ. Res..

[270] Crichton G.E., Alkerwi A. (2015). Physical activity, sedentary behavior time and lipid levels in the Observation of Cardiovascular Risk Factors in Luxembourg study. Lipids Health Dis..

[271] Zheng W., Chen Y., Zhao A., Xue Y., Zheng Y., Mu Z., Wang P., Zhang Y. (2016). Associations of sedentary behavior and physical activity with physical measurements and dyslipidemia in school-age children: A cross-sectional study. BMC Public Health.

[272] Ruiz-Ramie J.J., Barber J.L., Sarzynski M.A. (2019). Effects of exercise on HDL functionality. Curr. Opin. Lipidol..

[273] Vancheri F., Longo G., Vancheri E., Henein M.Y. (2022). Mental Stress and Cardiovascular Health-Part I. J. Clin. Med..

[274] Ajibewa T.A., Kershaw K.N., Carr J.J., Terry J.G., Gabriel K.P., Carnethon M.R., Wong M., Allen N.B. (2024). Chronic Stress and Cardiovascular Events: Findings From the CARDIA Study. Am. J. Prev. Med..

[275] Coughlin S.S. (2011). Post-traumatic Stress Disorder and Cardiovascular Disease. Open Cardiovasc. Med. J..

[276] Ryder A.L., Cohen B.E. (2021). Evidence for depression and anxiety as risk factors for heart disease and stroke: Implications for primary care. Fam. Pract..

[277] Andersen C.J., Walker B.G., Karanian T.J., Sloan A., Campbell C., Dupree L., Woodruff R. (2024). Diurnal patterns of salivary cytokines differentially correlate with greater fluctuations in cortisol and diet composition: A pilot study. J. Agr. Food Res..

[278] Shantakumari N., Sequeira S., El Deeb R. (2013). Effects of a yoga intervention on lipid profiles of diabetes patients with dyslipidemia. Indian Heart J..

[279] Booker J.M., Cabeza de Baca T., Trevino-Alvarez A.M., Stinson E.J., Votruba S.B., Chang D.C., Engel S.G., Krakoff J., Gluck M.E. (2024). Dietary Adherence Is Associated with Perceived Stress, Anhedonia, and Food Insecurity Independent of Adiposity. Nutrients.

[280] Sterrantino A.F. (2024). Observational studies: Practical tips for avoiding common statistical pitfalls. Lancet Reg. Health Southeast Asia.

